# FaMYB63 and FvWYRKY75 Activate *FvPR10.14* Boosting Strawberry Immunity Against Powdery Mildew

**DOI:** 10.1111/mpp.70186

**Published:** 2025-12-08

**Authors:** Rongyi Jiang, Tao Tao, Xingbin Xie, Yang Liu, Yanan Sun, Yang Zhang, Guanghui Zheng, Peipei Sun, Mauren Jaudal, Simona Nardozza, Congbing Fang, Jing Zhao

**Affiliations:** ^1^ School of Horticulture Anhui Agricultural University Hefei China; ^2^ Bioeconomy Science Institute Auckland New Zealand

**Keywords:** powdery mildew, PR10 gene, strawberry, transcription factor

## Abstract

Powdery mildew, caused by *Podosphaera aphanis*, poses a significant threat to strawberry production, while current chemical controls raise environmental and food safety concerns. In this study, we have identified the key regulatory module, FaMYB63/FvWRKY75‐*PR10.14*, that confers enhanced powdery mildew resistance in transgenic strawberry ( *Fragaria vesca* ). FaMYB63, an R2R3‐MYB transcription factor, was induced by *P. aphanis* infection and responsive to the application of defence signalling molecules, including salicylic acid (SA), methyl jasmonate (JA), abscisic acid (ABA), and 1‐aminocyclopropane‐1‐carboxylic acid (ACC). Silencing of *FaMYB63* led to reduced SA levels and increased powdery mildew susceptibility, accompanied by suppressed reactive oxygen species (ROS) bursts and down‐regulation of *PR10.14* expression. Conversely, overexpressing *PR10.14* in transgenic lines inhibited *P. aphanis* spore germination and enhanced ROS accumulation, indicating a dual role in direct pathogen inhibition and hypersensitive response‐triggered defence. Yeast one‐hybrid, electrophoretic mobility shift assay, β‐glucuronidase, and luciferase assays confirmed that FaMYB63 and FvWRKY75 were directly bound to the MYB‐binding sites and W‐box of the *PR10.14* promoter, respectively, and activated its transcription, while WRKY75 negatively regulated the expression of *MYB63*. *PR10.14* exhibited tissue‐specific expression, with the highest levels in red‐ripening fruits, suggesting a role in developmental‐stage‐dependent defence. These findings suggest FaMYB63 as an SA‐dependent regulator of *PR10.14*‐mediated resistance, bridging hormone signalling and pathogen response. This study provides a molecular target for breeding powdery mildew‐resistant strawberry cultivars through genetic engineering approaches, offering an alternative to fungicides for sustainable and environmental disease management in horticultural crops and advances our understanding of MYB‐ or WRKY‐PR10*P* networks in plant immunity.

## Graphical abstract

In response to powdery mildew infection, strawberry enhances disease immunity by activating *FvPR10.14* via a MYB63/WRKY75 transcriptional module through hormone‐ and ROS‐mediated signalling pathways.

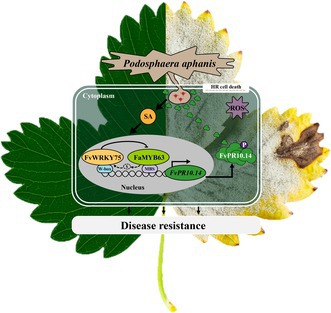

## 1 Introduction

Strawberry (*Fragaria × ananassa*) is one of the most widely grown and economically valuable berries globally due to its attractive colour, aroma, and nutritional properties (Fan et al. 2025; Newerli‐Guz et al. 2023). In 2023, world production and cultivated area of strawberries were approximately 10.5 million tonnes and 410,000 ha, respectively (FAOSTAT 2023). However, their productivity and postharvest quality are significantly constrained by biotic and abiotic stresses, including pathogen infections, pests, and suboptimal environmental conditions (Palmieri et al. 2017; Khammayom et al. 2022; Petrasch et al. 2019; Mozafari et al. 2022). Powdery mildew caused by *Podosphaera aphanis* (syn. *Sphaerotheca macularis*) has emerged as a devastating fungal disease in both open‐field and protected cultivation systems, severely threatening fruit quality and yield, thereby causing 20%–70% economic losses annually for growers in leading strawberry production regions worldwide (Aldrighetti and Pertot 2023; Heaven et al. 2023; Lynn et al. 2024). *P. aphanis*, either in the form of mycelium or chasmothecia, can thrive throughout the growth and developmental cycle of strawberry, primarily infecting leaves, petioles, flowers, peduncles, fruits, and even stolons. White mycelium typically appears on all aerial parts of infected plants, with young tissues being especially vulnerable, thereby disrupting photosynthetic and transpiration processes, which ultimately results in leaf necrosis and abscission (Jambagi and Dunwell 2015; Feng et al. 2020). The disease is favoured by moderate temperatures (15°C–25°C) and is characterised by high frequency, recurrence, rapid infection rate, and a short incubation period (Aldrighetti and Pertot 2023; Tapia et al. 2021).

Current powdery mildew management predominantly relies on synthetic fungicides targeting key fungal physiological processes, including sterol biosynthesis (triazoles), microtubule assembly (benzimidazoles), and amino acid metabolism (pyrimidines). These compounds exert their effects by inhibiting spore germination and the growth of fungal hyphae or infection structures (Hückelhoven 2005). Excessive application of fungicides raises concerns about environmental persistence and potential mycotoxin accumulation in edible plant tissues. This underscores the urgent need to develop powdery mildew‐resistant cultivars through molecular breeding, providing a long‐lasting strategy for disease control in ecofriendly agriculture.

Over the course of long‐term co‐evolution, natural selection has driven the development of sophisticated and complex defence mechanisms in plants at both the molecular and cellular levels in response to pathogens. The defence mechanisms of strawberries against pathogens operate through a multilayered protection system comprising three primary tiers: (1) passive defences, including structural barriers such as cuticular wax deposition, cell wall reinforcement, and constitutively produced antimicrobial compounds; (2) primary and secondary defence responses consisting of pattern‐triggered immunity (PTI) mediated by pathogen‐associated molecular patterns (PAMPs) and effector‐triggered immunity (ETI) mediated by R‐proteins; and (3) adaptive immune responses that coordinate a suite of defence mechanisms, including hypersensitive response (HR), reactive oxygen species (ROS) burst, phytohormonal signalling (e.g., salicylic acid [SA] and jasmonic acid [JA] pathways), and induction of pathogenesis‐related (PR) proteins (Amil‐Ruiz et al. 2011).

Pathogenesis Related Proteins 10 (PR10s) is a highly conserved protein family of small proteins (~154–163 amino acids) in angiosperms. The Bet v 1 domain is the three‐dimensional structure of most PR10 proteins, consisting of a seven‐stranded antiparallel β‐sheet, a short N‐terminal α‐helix, one long C‐terminal α‐helix and a P‐loop motif (Viboonjun and Longsaward 2024; Su et al. 2024).

PR10s play a role in developmental processes, secondary metabolism, and defence against pathogens and they have been extensively studied in several plant species with 18 identified proteins in apple, 17 in grape, 34 in common bean, 44 in banana, 34 in tea, and 6 in cashew (Gao et al. 2005; Bastiaan‐Net et al. 2020; He et al. 2013; Rajendram et al. 2022; Feki et al. 2024; Tao et al. 2025). In strawberry, the expression patterns of 21 *PR10* genes were investigated upon *Verticillium dahliae* infection (Besbes et al. 2019). Among them, four members exhibited significant up‐regulation in foliar tissues, while eight *PR10* genes were significantly up‐regulated in roots (Besbes et al. 2019). Notably, silencing the *PR10* gene *Fra a 1.02* in strawberry fruits did not affect anthocyanin biosynthesis during fruit development. Combined transcriptomic and metabolic profiling data indicated that *Fra a 1.02* might be involved in pathogen defence during fruit ripening (Orozco‐Navarrete et al. 2021). Nevertheless, the molecular interactions between PR10 and upstream transcription factors have not been elucidated in these previous studies.

PR10 proteins have DNase, RNase, and antibacterial activities due to their unique protein structure. For example, the recombinant VpPR10.2 protein has nuclease activity, which inhibited tobacco brown spot disease, and overexpression of *VpPR10.2* in susceptible grape cultivars enhanced their resistance to downy mildew (He et al. 2013). In banana, PR10 protein has β‐1,3‐glucanase and RNase activities, which can suppress the growth of the pathogenic fungus *Aspergillus fumigatus* (Rajendram et al. 2022). MdPR10‐1 and MdPR10‐2 have antibacterial activity in vitro and interacted with RNL2 and RNL6 to enhance resistance against apple Alternaria leaf spot (Zhang, Xu, et al. 2021). GmPR10 protein has RNase activity, and overexpression of *GmPR10* increased the resistance of tobacco and soybean to *Phytophthora nicotianae* and *Phytophthora sojae*, respectively (Xu et al. 2014). Moreover, overexpression of *OsPR10a* significantly enhanced the resistance of rice and *Arabidopsis* to *Xanthomonas oryzae* pv. *oryzae* and *Xanthomonas campestris* pv. *campestris* infections, respectively (Huang et al. 2016).

Transcription factors (TFs) are key regulatory proteins activating or inhibiting gene transcription by binding to specific target sequences or interacting with other associated proteins (Wang et al. 2016). MYB transcription factors constitute one of the largest and most multifunctional families in plants, playing critical roles in regulating various biological processes, including plant growth and development, secondary metabolism, hormone signal transduction, disease resistance, and responses to various biotic and abiotic stresses (Zheng et al. 2020; Tao et al. 2025). MYB TFs also play a critical role in the regulation of plant defence mechanisms against pathogens. For example, in cotton, GhMYB36, an R2R3‐type MYB, enhances tolerance to Verticillium wilt and drought stress by up‐regulating *PR1* expression (Liu et al. 2022). In grape, VqMYB154 mediates the plant's defence response to pathogens by activating the stilbenes pathway and subsequent production of ROS (Jiang et al. 2021). Moreover, VaMYB306 has been shown to interact with VaERF16, forming a transcriptional complex that binds to the promoter of *VaPDF1.2* and increases its transcript levels, thereby enhancing resistance to *Botrytis cinerea* (Zhu et al. 2022). In wheat, TaMYB29 positively regulates the plant's defence response against stripe rust by promoting H_2_O_2_ accumulation, PR gene expression, and cell death via the SA signalling pathway (Zhu et al. 2021). BrMYB108 confers resistance to Verticillium wilt by binding to the promoters of respiratory burst oxidase genes and enhancing their expression (Su et al. 2023). MdMYB116 interacts with MdbHLH093 to enhance apple resistance to powdery mildew by increasing H_2_O_2_ accumulation and activating the SA signalling pathway (Ma et al. 2023). FaMYB5, together with SA, positively regulates the biosynthesis of proanthocyanidins in strawberries, which are known to have antifungal properties and might play a role in the plant response to powdery mildew infections. However, the specific function of FaMYB5 in enhancing resistance to powdery mildew is unknown (Feng et al. 2020). As there is limited research on MYB TFs' role in regulating *PR10* genes in response to powdery mildew infection in strawberry, the mechanisms by which MYB TFs regulate powdery mildew resistance warrant further investigation.

As the largest family of transcriptional regulators in higher plants, WRKY TFs also play a critical role in modulating plant disease resistance responses (Wang, Wang, et al. 2023; Wang, Wu, et al. 2023; Pei et al. 2024). WRKY TFs initiate plant immune responses by recognising PAMPs and participate in downstream signal transduction pathways (Javed and Gao 2023). They regulate hormone signalling networks (including SA, JA and ethylene [ETH]), as well as the expression of downstream defence genes, by binding to the W‐box TGACC (A/T) in the promoters of their target genes (Pandey and Somssich 2009; Eulgem and Somssich 2007; Jiang, Ma, et al. 2017). Recently, *FaWRKY29* and *FaWRKY64* have been described as key susceptibility genes in strawberry, functioning through the regulation of abscisic acid (ABA) and JA signalling, cell wall modification, and ROS pathways in response to *B. cinerea* (Lee et al. 2023). MdWRKY71 acts as a susceptibility factor in apple Glomerella leaf spot by promoting SA degradation through activation of *MdDLO1*, thereby facilitating *Colletotrichum fructicola* infection (Pei et al. 2024). Overexpression of *CoWRKY78* suppresses anthracnose resistance in *Camellia oleifera* by disrupting ROS homeostasis and down‐regulating defence‐related genes in ROS homeostasis (Li et al. 2023). WRKY8 modulates ABA and ETH signalling pathways, mediating crosstalk that enhances the resistance of *Arabidopsis* to crucifer‐infecting tobacco mosaic virus (TMV‐cg) (Chen et al. 2013). Moreover, WRKY TFs interact with other TFs, including TGA and NPR1, to form complex regulatory networks that enhance pathogen recognition and resistance (Pandey and Somssich 2009; Jiang, Ma, et al. 2017). OsWRKY6 binds directly to the W‐box‐like element 1 (WLE1) in the *OsPR10a* promoter and positively activates *OsPR10a* expression, enhancing resistance to pathogens (Choi et al. 2015). There are 64 and 257 WRKY TFs in diploid woodland and octoploid strawberries (Garrido‐Gala et al. 2022), respectively; however, to date, there is no report to functionally validate a *PR10* gene in strawberry as a direct target of WRKY TFs.

We observed that our FaMYB63‐RNAi transgenic strawberry lines (Wang, Shi, et al. 2022) appeared more susceptible to powdery mildew when compared to wild type (WT) plants and thus represent an ideal model to study the involvement of FaMYB63 in powdery mildew defence in strawberries. We hypothesised that FaMYB63 is a key regulator in the resistance mechanism to powdery mildew and it binds directly to an unknown *PR10* gene. Our results revealed that high SA induced the expression of the *PR10* gene *FvPR10.14*, which was identified as a positive regulator enhancing resistance to powdery mildew in transgenic strawberry. We uncovered a function for FaMYB63, which directly binds to the promoter of *FvPR10*.*14*, and could be important in regulating powdery mildew resistance. In this study, we discuss the novel role of the FaMYB63/FvWRKY75‐*FvPR10.14* regulatory module and the relationship between SA and powdery mildew and offer new opportunities for genetic improvement of strawberry resistance to powdery mildew.

## 2 Results

### 2.1 Down‐Regulation of *FaMYB63* Increases the Susceptibility of Strawberry Plants to *P. aphanis*

**FIGURE 1:**
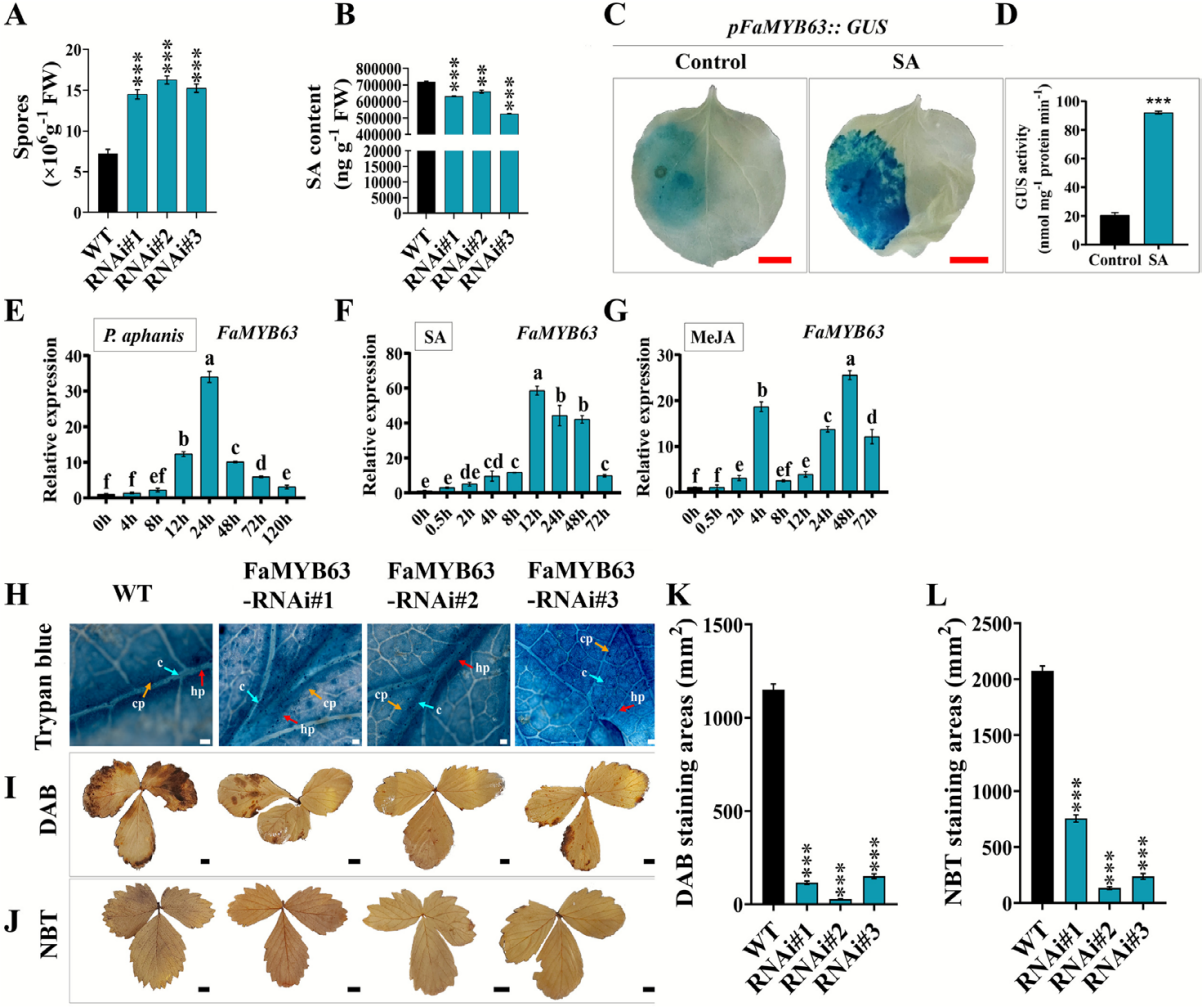
Induction of FaMYB63 by hormonal treatments and *Podosphaera aphanis* infection. (A) Quantification of conidia on wild‐type (WT) and FaMYB63‐RNAi plants (RNAi#1, #2, and #3) at 7 days post‐inoculation (dpi) with *P. aphanis*. (B) Salicylic acid (SA) concentrations in FaMYB63‐RNAi transgenic and WT plants. (C and D) Histochemical staining for β‐glucuronidase (GUS) (C) and GUS activity (D) of the *FaMYB63* promoter. (E–G) Time‐course expression of *FaMYB63* following *P. aphanis* inoculation (E), SA treatment (F), and methyl jasmonate (MeJA) treatment (G). (H) Trypan blue staining of leaves of WT and RNAi‐FaMYB63 lines at 7 dpi with *P. aphanis*. c, conidium; hp, hypha; cp, conidiophore. (I, J) 3,3′‐diaminobenzidine (DAB) (I) and nitroblue tetrazolium (NBT) (J) staining of leaves of WT and RNAi‐FaMYB63 lines at 7 dpi with *P. aphanis*. (K) DAB and (L) NBT staining areas were quantified by ImageJ software. Red scale bar = 1 cm, white scale bar = 200 μm; black scale bar = 5 mm. Data indicates mean ± standard deviation for three replicates. Different letters indicate significant difference at *p* < 0.05 (Tukey's HSD test). Student's *t* test: ***p < 0.001, **p < 0.01.

Three FaMYB63‐RNAi transgenic strawberry lines (Figure S1) were previously obtained (Wang, Shi, et al. 2022). Phenotypic observation suggested that they were more susceptible to powdery mildew than the WT under normal environmental conditions. To confirm these qualitative results, we infected strawberry leaves with *P. aphanis* and found that the FaMYB63‐RNAi lines had large white hyphal areas on leaves, while the WT strain had small white fungal spots. The number of spores on the leaves was collected and counted (Figure 1A). The three FaMYB63‐RNAi transgenic lines exhibited significantly higher conidial numbers compared with the WT (*p* < 0.001), with increases of 100%, 124%, and 110%, respectively. Thus, the transgenic lines had a more severe disease incidence than the WT, indicating that silencing of *FaMYB63* reduced the resistance of strawberries to powdery mildew.

### 2.2 FaMYB63 Responds to *P. aphanis* Infection and Hormone Treatments

The FaMYB63‐RNAi transgenic lines produced significantly less SA than the WT when grown under normal conditions (Figure 1B; *p* < 0.01). We also examined the expression of the SA pathway‐related genes in the FaMYB63‐RNAi transgenic lines and found that transcription of the SA biosynthetic genes *FvICS1* and *FvPAL2* was significantly decreased, while the expression of *FvPR1*, *FvPR2‐1* and *FvPR5‐2* increased (Figure S2). In a transient assay in *Nicotiana benthamiana* leaves in which the promoter of *FaMYB63* was fused to the β‐glucuronidase gene *GUS* (*pFaMYB63*::GUS), SA treatment promoted GUS activity (Figure 1C,D). To determine whether *FaMYB63* expression was induced by *P. aphanis*, SA, or MeJA treatments, a reverse transcription‐quantitative PCR (RT‐qPCR) assay was performed using WT strawberry seedlings treated with *P. aphanis*, SA or MeJA. *FaMYB63* showed a similar pattern of expression after *P. aphanis* and SA treatments, being up‐regulated and reaching the highest levels at 24 h and 12 h, respectively (Figure 1E,F). *FaMYB63* was also up‐regulated by MeJA treatment; however, it exhibited biphasic expression peaks at 4 h and 48 h (Figure 1G). These results indicated that the induction of *FaMYB63* expression by *P. aphanis* is consistent with activation of defence‐related signalling, potentially mediated by SA and/or MeJA.

To further investigate whether the disease resistance response mediated by *FaMYB63* is associated with the accumulation of ROS, leaves from the FaMYB63‐RNAi transgenic lines and WT plants were subjected to 3,3′‐diaminobenzidine (DAB) and nitroblue tetrazolium (NBT) staining after 7 days of inoculation with *P. aphanis*. In addition, hyphal development was assessed using trypan blue staining. Trypan blue staining revealed that the mycelia and conidia on WT leaves were fewer than those on FaMYB63‐RNAi transgenic lines and primarily distributed around the leaf veins (Figure 1H). Moreover, we found significant decreases (*p* < 0.001) in the intensity and area of DAB (H_2_O_2_) and NBT (ROS) staining in FaMYB63‐RNAi transgenic lines compared to WT. After infection of strawberry leaves by *P. aphanis*, the burst of ROS in the FaMYB63‐RNAi transgenic lines was affected (Figure 1I–L). These results indicated that powdery mildew developed faster on the leaves of FaMYB63‐RNAi transgenic lines compared with WT.

### 2.3 RT‐qPCR Analysis of *PR10* Genes in Response to *P. aphanis* Infection

**FIGURE 2:**
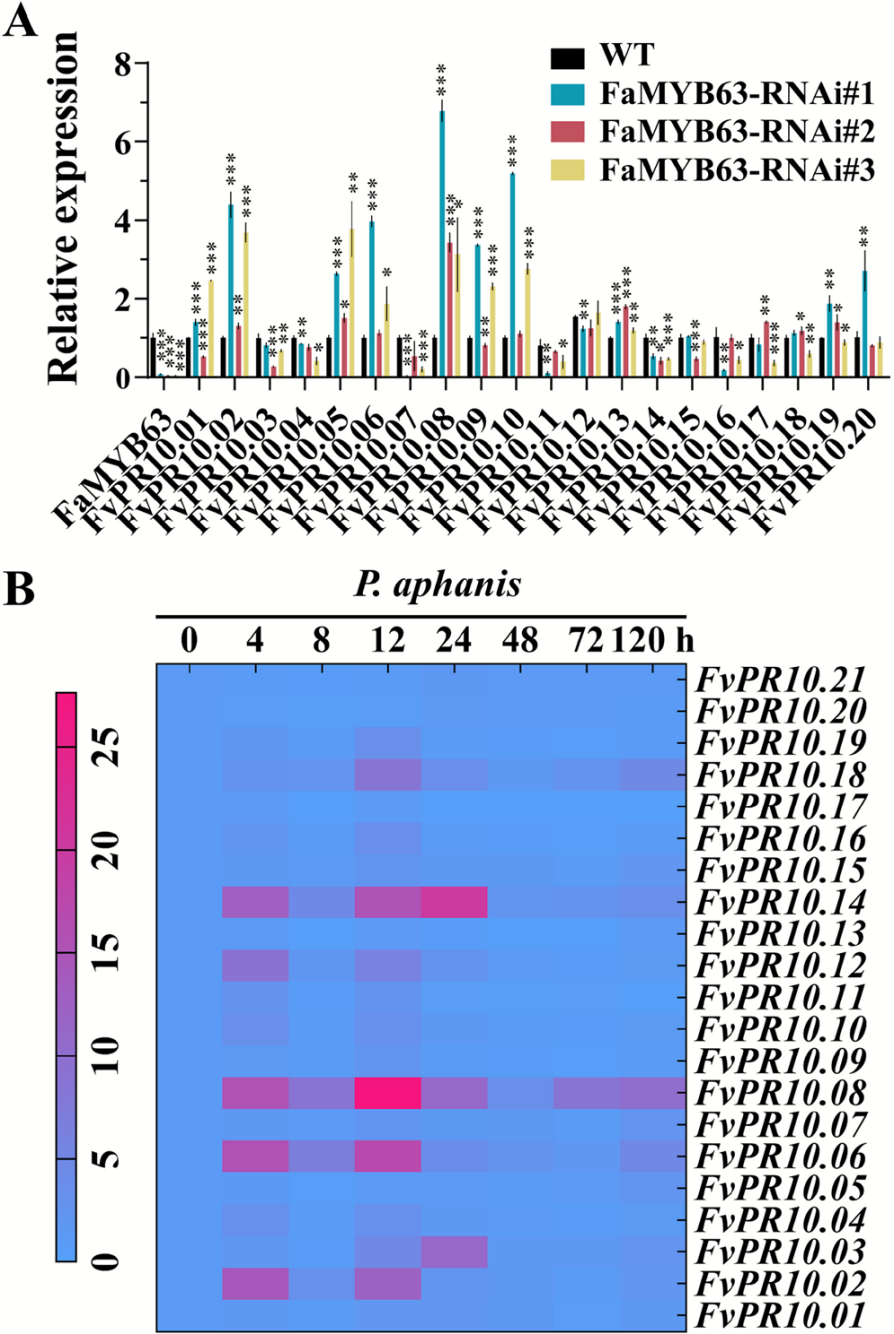
The expression levels of *PR10* genes in FaMYB63‐RNAi transgenic plants and during *Podosphaera aphanis* infection. (A) Transcript levels of *PR10* genes in wild‐type (WT) and RNAi‐FaMYB63 transgenic plants. (B) Expression patterns of *PR10* genes after inoculation with *P. aphanis*. The colour scale represents relative expression levels, with red indicating increased transcript abundance and blue indicating decreased transcript abundance. Error bars are SD of three biological replicates. Asterisks represent significant differences from WT: ***p < 0.001, **p < 0.01, *p < 0.05.

Twenty‐one *Pathogenesis Related Proteins 10* (*FaPR10*) genes from the octoploid strawberry were previously isolated and shown to play a role in defence response to *V. dahliae* (Besbes et al. 2019). Therefore, we analysed the transcript abundance of the 21 homologous *FvPR10* genes in the FaMYB63‐RNAi transgenic lines and their response to *P. aphanis* infection. The results showed that *FvPR10.03*, *FvPR10.07*, *FvPR10.11*, *FvPR10.14* and *FvPR10.16* were down‐regulated in the FaMYB63‐RNAi transgenic lines (Figure 2A). After inoculation with *P. aphanis*, seven *FvPR10* genes were significantly up‐regulated, with *FvPR10.03*, *FvPR10.08* and *FvPR10.14* up‐regulated by 11–28‐fold (Figure 2B). The transcription patterns of the *FvPR10* genes after SA, MeJA, 1‐aminocyclopropane‐1‐carboxylic acid (ACC, the direct precursor of ETH), and ABA treatments were also investigated; 13 *FvPR10* genes were up‐regulated and three *FvPR10* genes were down‐regulated. Among them, only the expression of *FvPR10.14* was significantly up‐regulated by SA treatment (Figure S3). Based on these results, we selected *FvPR10.14* for further investigation to understand its involvement in strawberry resistance to powdery mildew.

### 2.4 FaMYB63 Binds to the *PR10.14* Promoter and Activates Its Transcription

Similar to *FaMYB63*, *FvPR10.14* was significantly up‐regulated by both *P. aphanis* infection and SA treatment. Among the *FvPR10* genes, *FvPR10.14* was consistently down‐regulated in the three FaMYB63‐RNAi transgenic lines (Figure 2). We therefore hypothesise a possible regulation of *FvPR10.14* by FaMYB63 to positively regulate strawberry resistance to powdery mildew.

**FIGURE 3:**
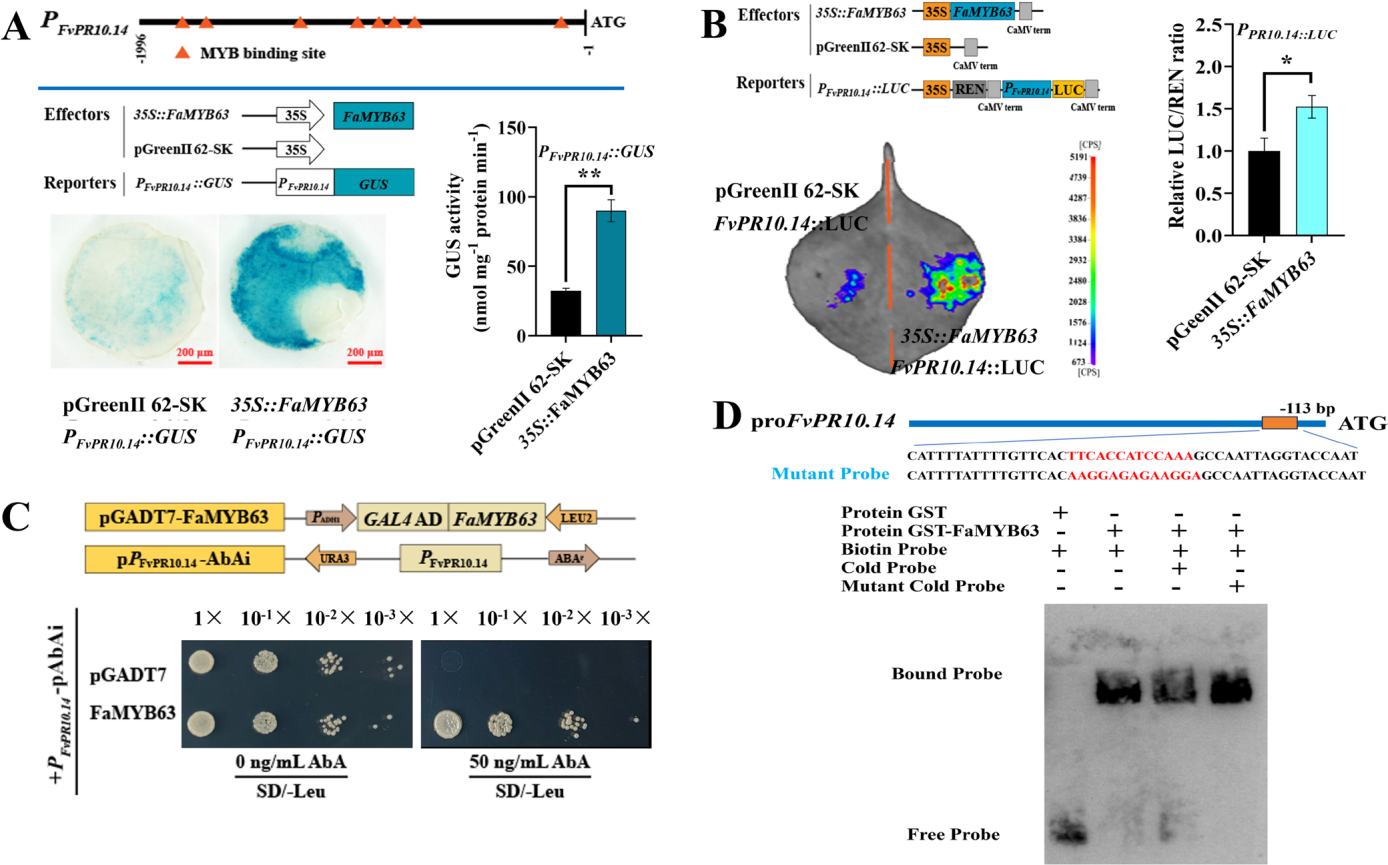
FaMYB63 positively regulates the expression of *FvPR10.14* by directly binding to its promoter. (A) β‐Glucuronidase (GUS) reporter assay showed that FaMYB63 positively regulates *FvPR10.14* expression by binding to its promoter. Red scale bar = 200 μm. (B) Dual‐luciferase (LUC) assay further validated that FaMYB63 activates *FvPR10.14* promoter activity. The data in (A, B) are presented as the mean ± SD (*n* = 9). Asterisks indicate significant difference from the control using Student's *t* test: **p < 0.01, *p < 0.05. (C) Yeast one‐hybrid assay demonstrated the direct binding of FaMYB63 to the *FvPR10.14* promoter. (D) Electrophoretic mobility shift assay showed binding of FaMYB63 to the *FvPR10.14* promoter region in vitro. Probe sequences of *FvPR10.14* promoter region and mutant versions of the *FvPR10.14* promoter are shown at the top. ‘+’ and ‘−’ indicate the presence or absence of the probe or protein, respectively.

As FaMYB63 is a R2R3‐MYB TF that recognises and binds to the MYB binding site (MBS) in its target promoters (Millard et al. 2019), the sequences of the *FvPR10.14* promoter were analysed, and eight MBS motifs were identified (Figure 3A, Figure S4). GUS and luciferase (LUC) assays revealed that FaMYB63 positively regulated *FvPR10.14* activity by binding to its promoter (Figure 3A,B). To further investigate whether FaMYB63 could bind directly to the promoter of *FvPR10.14*, a yeast one‐hybrid (Y1H) assay was carried out. The assay revealed that the yeast cells co‐transformed with FaMYB63‐effector and pro*FvPR10.14*‐reporter grew well on SD/−Leu medium, indicating direct binding of FaMYB63 to pro*FvPR10.14* (Figure 3C). Electrophoretic mobility shift assay (EMSA) results also showed that FaMYB63 specifically bound to the *FvPR10.14* promoter (Figure 3D). The binding of FaMYB63 to the *FvPR10.14* promoter was distinctly reduced after a competitive probe was added, whereas the binding of FaMYB63 was not affected by the addition of a competitive probe with mutated nucleotides. These results suggest that FaMYB63 is an activator of *FvPR10.14*, which might play an important role in strawberry resistance to powdery mildew.

### 2.5 Characterisation of *FvPR10.14*

**FIGURE 4:**
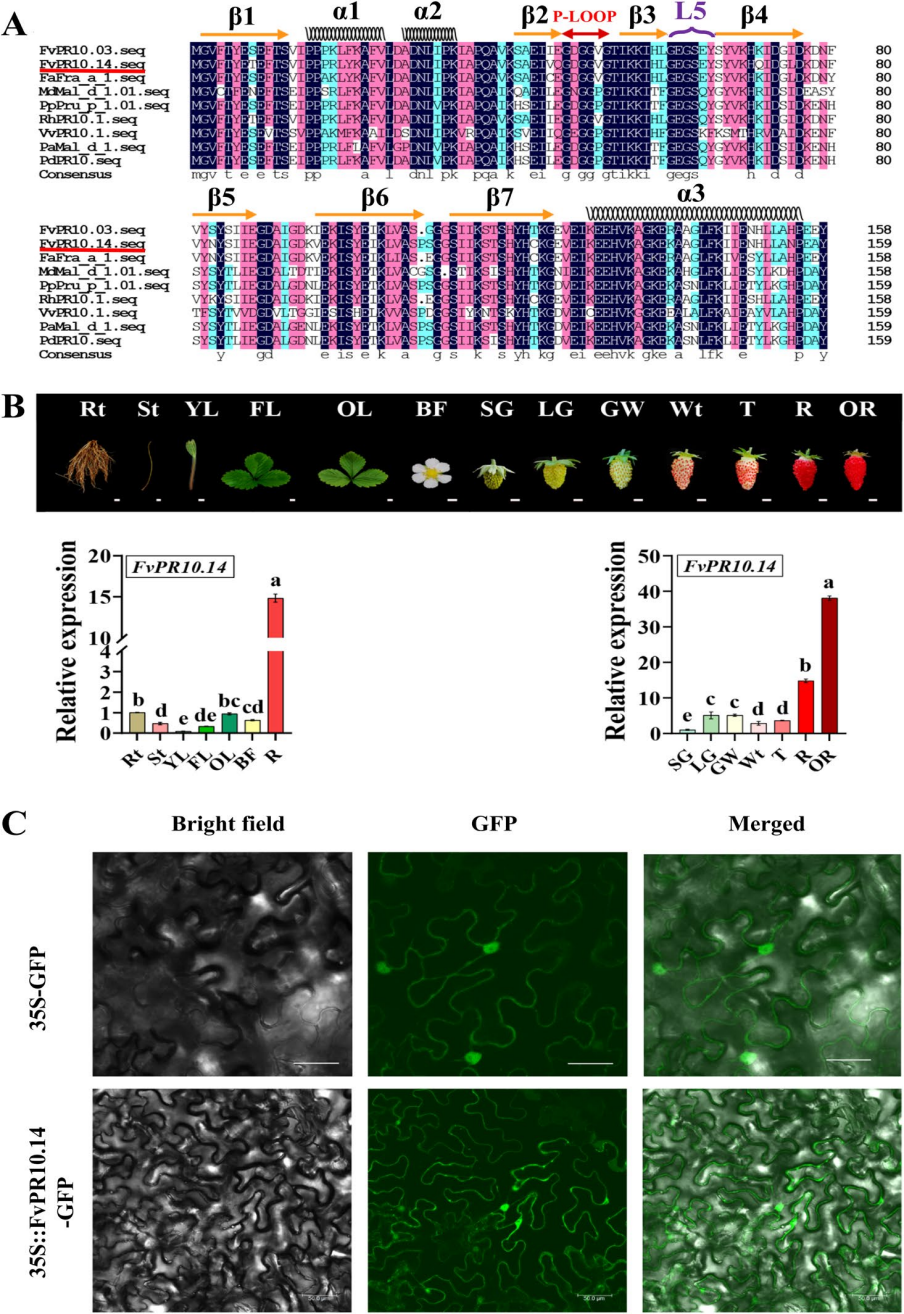
Characterisation of *FvPR10.14*. (A) Comparison of *Fragaria vesca* FvPR10.14 with homologous proteins from other plant species. (B) Transcript abundance of *FvPR10.14* in different tissue organs and developmental stages of strawberry. Root (Rt), stolon (St), young leaves (YL), functional leaves (FL), old leaves (OL), blooming flower (BF), small green fruit stage (SG), large green fruit stage (LG), green‐white fruit stage (GW), white fruit stage (Wt), turning fruit stage (T), red‐ripening fruit stage (R), and over‐ripening stage (OR). (C) Subcellular localisation of FvPR10.14. Bar = 50 μm. Data are expressed as mean ± SD of three biological replicates. Different letters above bars mean significant differences from each other at *p* < 0.05 (Tukey's HSD test).

The full‐length open reading frame (ORF) of *FvPR10.14* is 483 bp and encodes 161 amino acids. The protein structure, analysed by using STMART and Pfam, consists of three α‐helices, seven β‐sheets, and one P‐loop, which is a classical PR10 family Bet_V1 domain structure (Figure 4A). The FvPR10.14 protein clustered with octoploid strawberry (FaFra a 1.08) and rose (RhPR10.1) proteins (Figure S5). RT‐qPCR results showed that *FvPR10.14* was highly expressed in roots (Rt) and red‐ripening fruits (R), but lowest in young leaves (YL) (Figure 4B). The transcript abundance of *FvPR10.14* increased during strawberry fruit development and reached the highest amount at the over‐ripening (OR) fruit stage (Figure 4B). Subcellular localisation revealed that FvPR10.14 was not limited to the cytoplasm but was also observed in the nucleus (Figure 4C).

**FIGURE 5:**
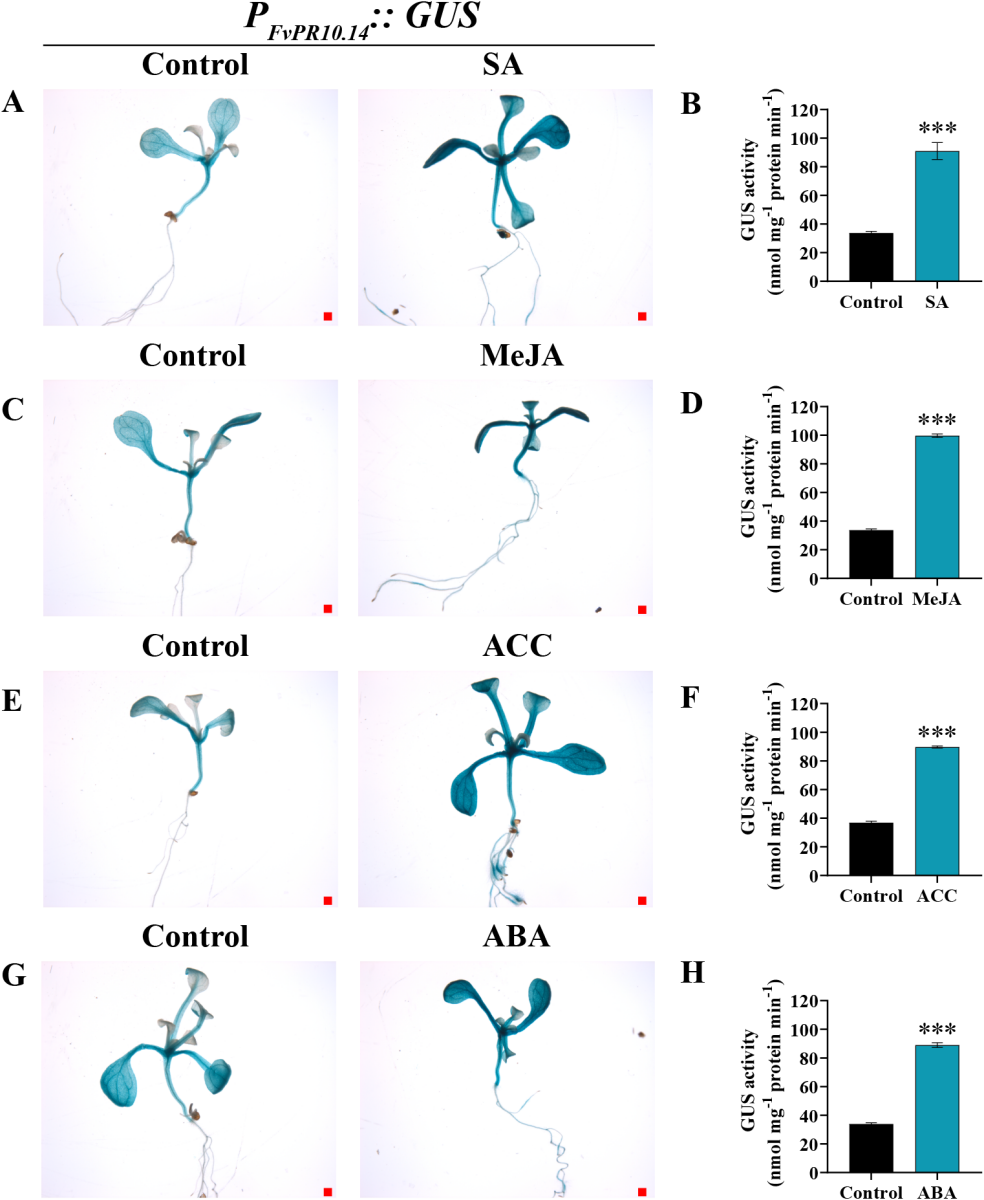
Hormone‐induced *P*_*FvPR10.14*_ *∷GUS* expression and activity in transgenic *Arabidopsis*. (A, C, E, G) Histochemical staining for β‐glucuronidase (GUS) of transgenic *Arabidopsis* harbouring the *P*_*FvPR10.14*_ *∷GUS* construct before (control) and after hormone treatments: salicylic acid (SA), methyl jasmonate (MeJA), 1‐aminocyclopropane‐1‐carboxylic acid (ACC), and abscisic acid (ABA). (B, D, F, H) Fluorometric GUS activity assay of *P*_*FvPR10.14*_ *∷GUS* plants before and after SA, MeJA, ACC and ABA treatments. Bar = 100 μm. Error bars indicate SD (*n* = 9). Significant differences from the control (***p < 0.001) were determined by Student's *t* test.

Promoter analysis of *FvPR10.14* identified multiple *cis*‐regulatory elements associated with defence responses and TF binding. The promoter harbours eight MYB binding sites, three MYC binding sites, five W boxes (WRKY binding sites), one TCA‐element (SA‐responsive), three ABREs (ABA‐responsive), one ERE (ethylene‐responsive), and one TCT‐motif (light‐responsive), suggesting that *FvPR10.14* transcription may be coordinately regulated by multiple defence‐ and hormone‐related pathways (Figure S4). To verify whether hormone signalling affects the activity of the *FvPR10.14* promoter (Figure 5), transgenic *Arabidopsis* with P_*FvPR10.14*_::GUS construct was treated with 0.1 mM SA, 0.1 mM MeJA, 0.1 mM ACC or 0.1 mM ABA. All four hormone treatments enhanced the expression and activity of P_*FvPR10.14*_::GUS, resulting in an increase of 170%, 195%, 144% and 163%, respectively (Figure 5).

### 2.6 Overexpression of *FvPR10.14* Enhances Powdery Mildew Resistance in *Fragaria vesca*

**FIGURE 6:**
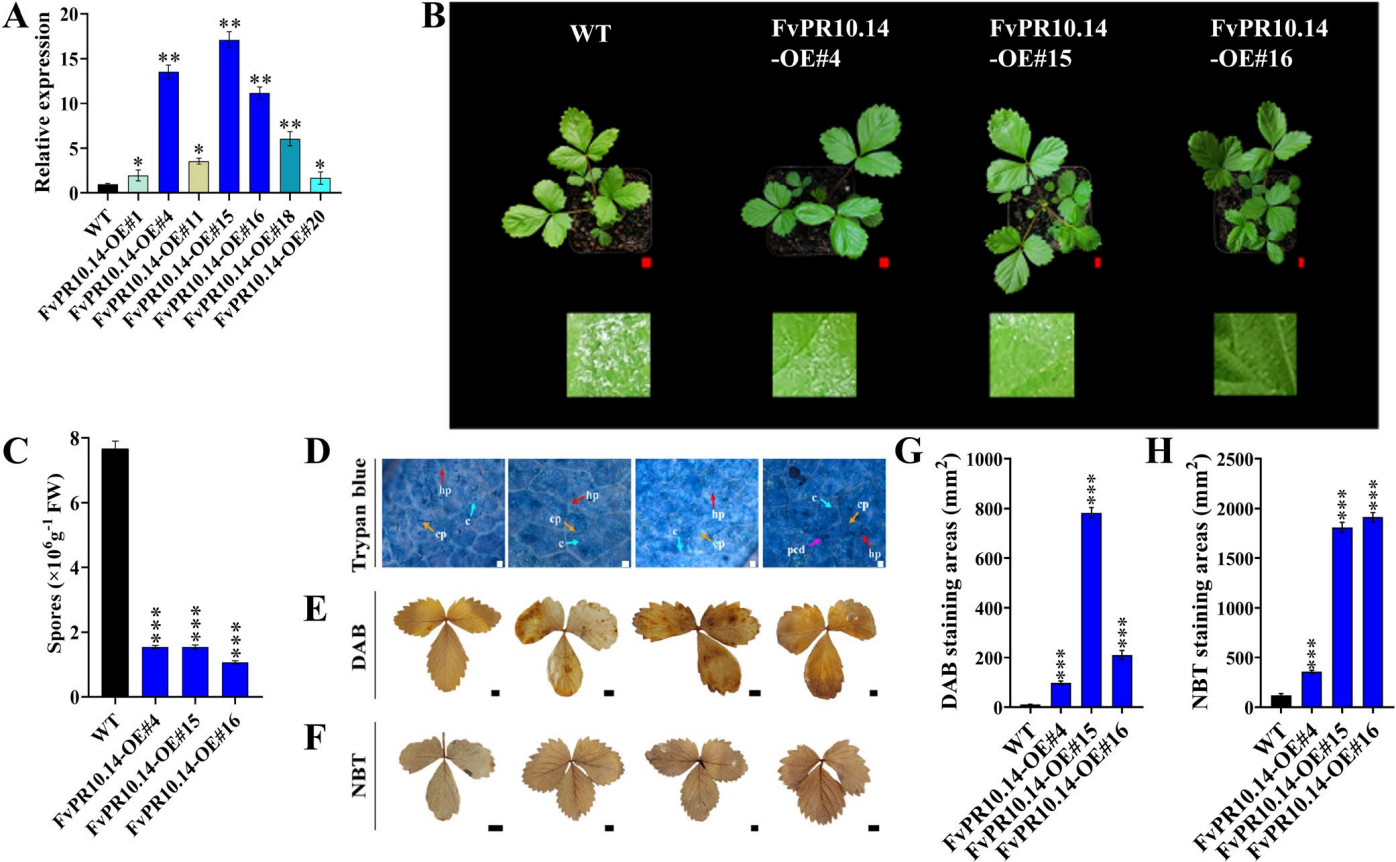
Transgenic overexpression of *FvPR10.14* in strawberry enhances powdery mildew resistance. (A) Gene expression level of *FvPR10.14* in seven FvPR10.14‐OE transgenic *Fragaria vesca* lines compared to the wild type (WT). (B) Disease phenotype of the *FvPR10.14*‐overexpressing strawberry plants at 7 days post‐inoculation (dpi) with *Podosphaera aphanis*. (C) Quantification of conidia in *FvPR10.14*‐OE plants at 7 dpi. (D) Trypan blue staining of *FvPR10.14*‐OE strawberry leaves at 7 dpi. c, conidium; hp, hypha; cp, conidiophore; pcd, programmed cell death. (E) 3,3′‐diaminobenzidine (DAB) staining of *FvPR10.14*‐OE strawberry leaves at 7 dpi. (F) Nitroblue tetrazolium (NBT) staining of *FvPR10.14*‐OE strawberry leaves at 7 dpi. The areas of DAB (G) and (H) NBT staining were quantified using ImageJ software. White bar = 500 μm; red and black bars = 5 mm. Data are means ± SD (*n* = 3). Asterisks indicate significant differences from WT by Student's *t* test: ***p < 0.001, **p < 0.01, *p < 0.05.

To investigate the role of *FvPR10.14* in *P. aphanis* infection in strawberry, the *35S::FvPR10.14*::GFP construct was transformed into the diploid strawberry *F. vesca* ‘Ruegen’ using *Agrobacterium* *tumefaciens* ‐mediated transformation. Seven independent transgenic lines with up‐regulated *FvPR10.14* expression were obtained (Figure 6A). RT‐qPCR revealed that the transcripts of *FvPR10.14* in the FvPR10.14‐OE#4, ‐OE#15, and ‐OE#16 lines were up‐regulated by 11 to 16‐fold compared to WT and overexpression of the transgenes was also confirmed by GFP fluorescence in the roots (Figure S6). The three overexpressing lines were selected for further analysis (Figure 6B).

To determine whether overexpression of *FvPR10.14* in transgenic strawberries can affect powdery mildew resistance, *P. aphanis* was inoculated onto the plant leaves. The overexpressing lines were significantly less susceptible to powdery mildew than WT and presented only a small amount of powdery mildew spots appearing on young leaves after 7 days. Moreover, the number of conidia on the infected leaves of the three transgenic lines decreased by 80%, 80%, and 86%, respectively, compared with the WT (Figure 6C). Trypan blue staining showed that the number of germinated conidia on the leaf surface of WT plants was significantly higher than in *FvPR10.14*‐overexpressing transgenic plants, with more new conidia produced, starting a new round of infection. In contrast, the *FvPR10.14*‐overexpression lines exhibited significantly slower hyphal growth, with reduced development of secondary hyphae and conidia (Figure 6D).

DAB staining showed that the leaves of *FvPR10.14*‐overexpression lines produced more H_2_O_2_ than the WT (*p* < 0.001; Figure 6E,G). NBT staining further confirmed this result, as transgenic plants accumulated higher concentrations of O_2_^·‐^ near the leaf veins than WT plants (*p* < 0.001; Figure 6F,H). These results suggest that *FvPR10.14* is involved in the response triggered by *P. aphanis*, which in turn inhibits the proliferation of the fungus.

### 2.7 WRKY75 Represses MYB63 Independently of Physical Interaction

**FIGURE 7:**
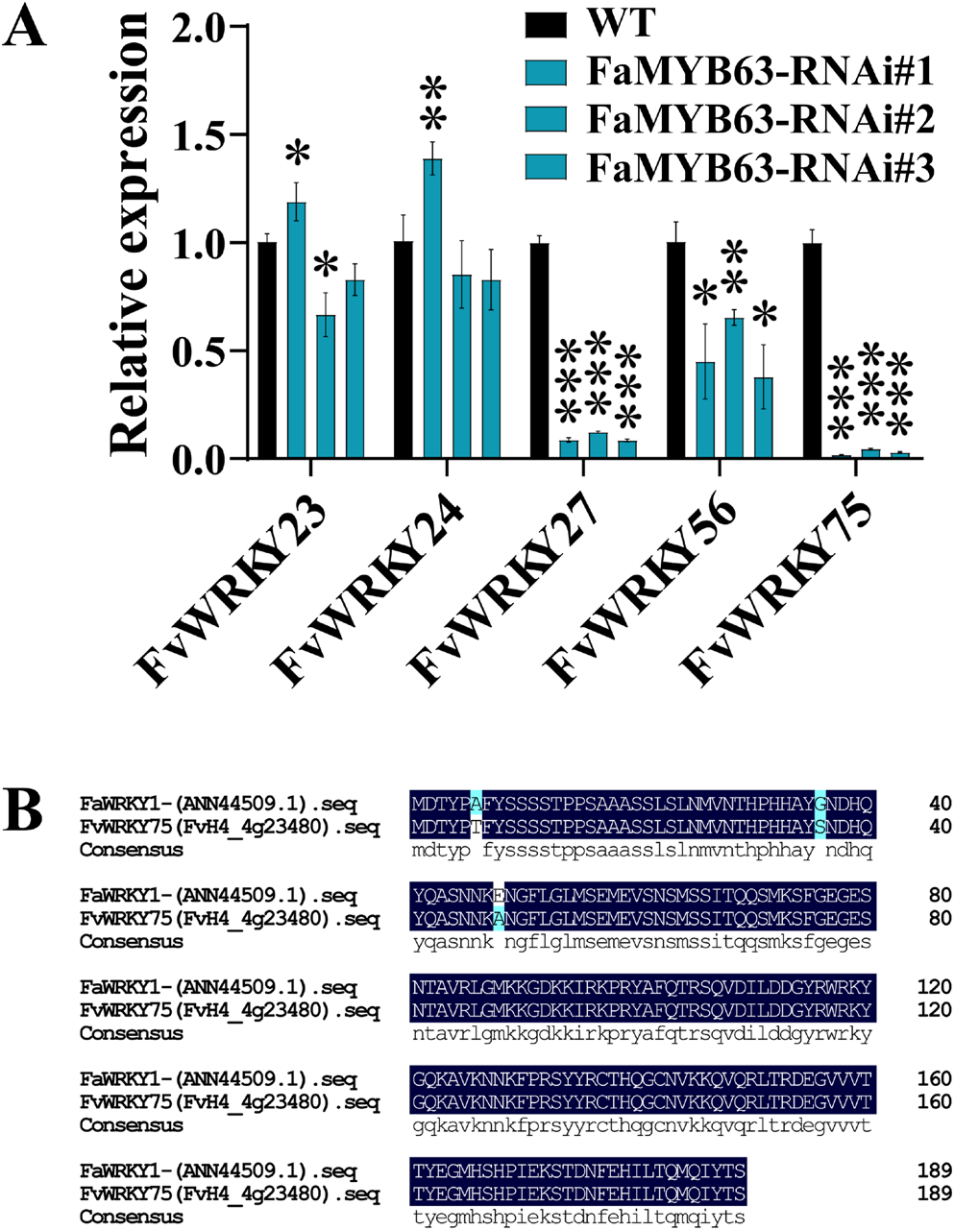
Analysis of *FvWRKYs* expression levels in FaMYB63‐RNAi strawberry plants. (A) *FvWRKYs* transcripts in FaMYB63‐RNAi lines. (B) Amino acid sequence alignment between FvWRKY75 and FaWRKY1. Error bars indicate SD (*n* = 3). Asterisks indicate significant differences from the wild type (WT) using Student's *t* test: ***p < 0.001, **p < 0.01, *p < 0.05.

WRKY TFs are widely known as key regulators of plant defence responses. To explore whether WRKY proteins participate in the FaMYB63*‐PR10.14* regulatory pathway in strawberry, we screened WRKY genes previously reported to function in disease resistance in strawberry. Using RT‐qPCR analysis, we found that the expression levels of *FvWRKY27*, *FvWRKY56*, and *FvWRKY75* were all reduced in the FaMYB63‐RNAi transgenic lines, with *FvWRKY75* showing the most significant down‐regulation (Figure 7). The *FvWRKY75* gene has a full‐length coding sequence of 573 bp, encoding 191 amino acids. It shares a high degree of sequence similarity with *FaWRKY1* from the octoploid cultivated strawberry, differing by only three amino acids. FaWRKY1 acts as a positive regulator and plays a key role in strawberry defence against *Colletotrichum acutatum* (Encinas‐Villarejo et al. 2009). To investigate potential regulatory interactions, we assessed if MYB63 and WRKY75 TFs interact with the promoters of *WRKY75* and *MYB63,* respectively, and whether a complex interaction network is present. We showed that *FvWRKY75* expression was not directly activated by FaMYB63 (Figure S7). We then divided the *MYB63* promoter into two fragments (P1 and P2, Figure S8A) and found that P2 was bound to WRKY75, while P1 was not. Moreover, WRKY75 significantly repressed *MYB63* expression (*p* < 0.001; Figure S8B). To test the interaction between the two proteins, we carried out a split luciferase complementation assay (Split‐LUC) and a yeast two‐hybrid (Y2H) assay. As shown in Figure S9A, no LUC fluorescence signal was detected in *N*. *benthamiana* leaves co‐infiltrated with MYB63‐cLUC and WRKY75‐nLUC. Consistently, the Y2H assay confirmed that MYB63 did not interact with WRKY75 (Figure S9B).

**FIGURE 8:**
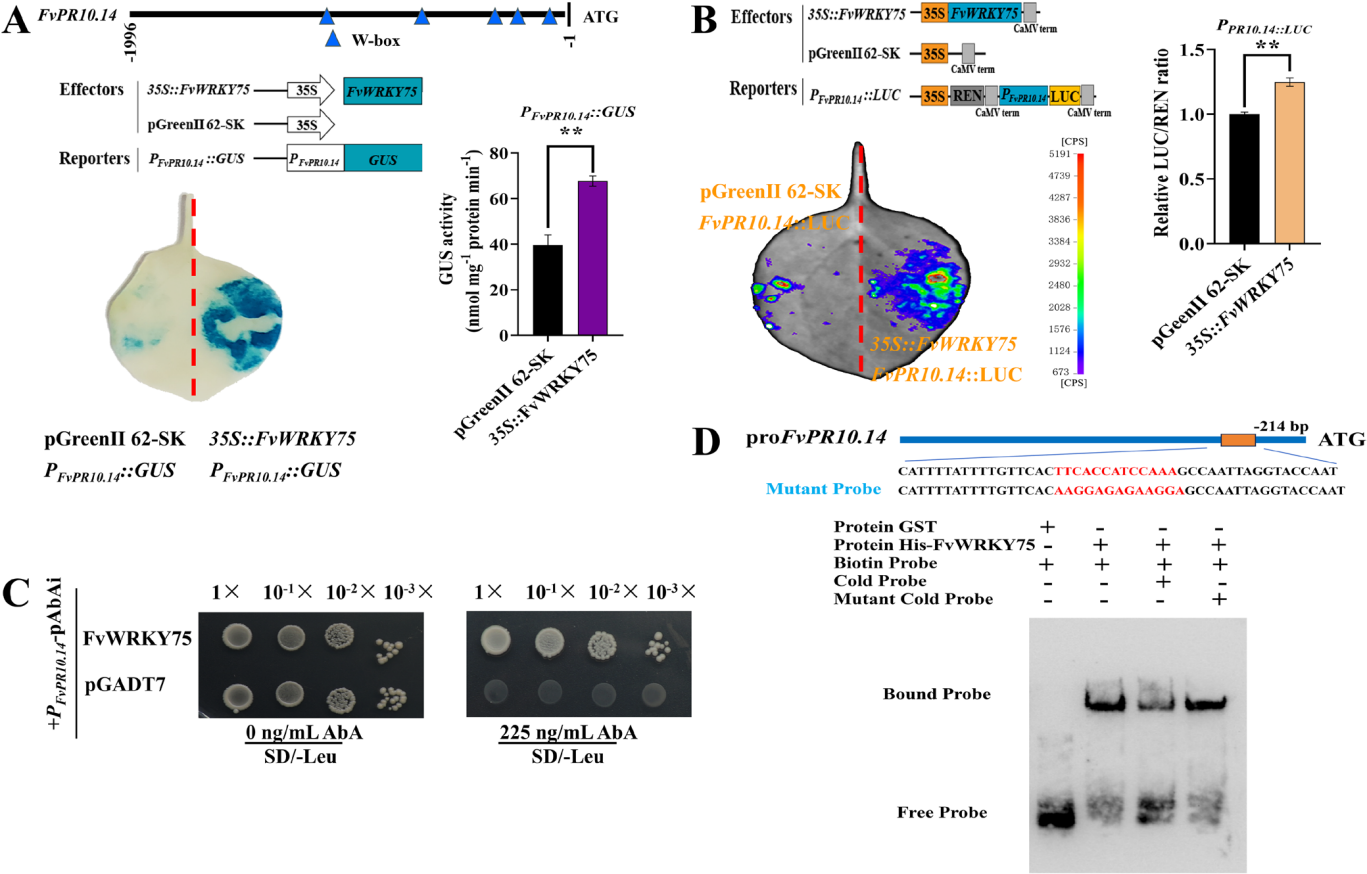
FvWRKY75 is bound to the *FvPR10.14* promoter and positively regulates its expression. (A) β‐glucuronidase (GUS) assays confirmed that FvWRKY75 positively regulates *FvPR10.14* expression by binding to its promoter. The data are presented as the mean ± SD (*n* = 9). (B) Dual‐luciferase (LUC) assays further validated that FvWRKY75 increased *FvPR10.14* promoter luminescence intensity. Data are means ± SD of nine biological replicates. Asterisks indicate significant differences from the control by Student's *t* test, **p < 0.01. (C) Yeast one‐hybrid assays showed the direct binding of FvWRKY75 to the *FvPR10.14* promoter. (D) Electrophoretic mobility shift assay of the binding of FvWRKY75 to the promoter of *FvPR10.14*. Probe sequences of the *FvPR10.14* promoter region and mutant versions of the *FvPR10.14* promoter are shown at the top. ‘+’ and ‘−’ indicate the presence or absence of the probe or protein, respectively.

To verify whether FvWRKY75 regulates *FvPR10.14* expression, LUC and GUS staining assays were performed (Figure 8). Compared to the empty vector, GUS activity increased by approximately 1.75‐fold when FvWRKY75 and *P*_*FvPR10.14*_ were co‐transformed (Figure 8A). LUC activity assays further demonstrated that FvWRKY75 up‐regulated the expression of *FvPR10.14* and enhanced its promoter activity by about 1.25‐fold, indicating that FvWRKY75 also positively regulates *FvPR10.14* (Figure 8B). EMSA and Y1H assays were conducted to investigate the interaction between the *FvPR10.14* promoter and FvWRKY75. In contrast to the control, the yeast strain co‐transformed with pGADT7‐FvWRKY75 and pro*FvPR10.14* grew on SD/−Leu medium supplemented with 225 ng/mL aureobasidin A, indicating that FvWRKY75 binds to the *FvPR10.14* promoter (Figure 8C). EMSA further confirmed this result (Figure 8D).

## 3 Discussion

Powdery mildew is a devastating fungal disease that limits crop yields and compromises the nutritional quality of fruits. To counteract this biotic stress, plants have evolutionarily developed multilayered regulatory mechanisms integrating phytohormone signalling, epigenetic modifications, and TF‐mediated surveillance systems across diverse ecological niches (Amil‐Ruiz et al. 2011; Liu et al. 2022; Jiang et al. 2021). Identification of important genes in the strawberry defence response to *P. aphanis* is critical for breeding new cultivars resistant to powdery mildew.

Unlike most PR proteins, the PR10 protein family is a large group that lacks signal peptides and is typically located in the cell nucleus, membrane, and cytoplasm (Guo et al. 2022; Tao et al. 2025). We showed that FvPR10.14 was localised in the cell membrane and nucleus. The PR10 protein family plays an important role in pathogen response, due to its RNase, DNase, and antibacterial activities, in many plant species including rice (Li et al. 2022), grape (Ma et al. 2018), pepper (Choi et al. 2012), pear (Su et al. 2024), and apple (Zhang, Xu, et al. 2021). *PR10* genes exhibit considerable variability in their induction patterns across plant species. For instance, in sugarcane, *ScPR10* is strongly up‐regulated by SA and MeJA (Peng et al. 2017). The grape *VpPR10.02* responds to SA, MeJA, ABA, cold, drought, and heat stress (He et al. 2013). In *Phaseolus vulgaris* , *PvPR10* genes display distinct expression profiles under biotic and abiotic stress (Feki et al. 2024). The rice *RSOsPR10* is induced by JA, drought, and salt stress, remains unresponsive to low temperature and ABA, and notably, is suppressed by SA (Takeuchi et al. 2016). In alfalfa, *MsPR10.1A* and *MsPR10.1B* are up‐regulated by ETH and ABA (Bahramnejad et al. 2010). Despite their implication in plant defence, the biological roles of PR10 proteins in disease resistance and their upstream regulatory TFs remain largely elusive, particularly in strawberry. In this study, the expression of *PR10.14* was significantly down‐regulated in the FaMYB63‐RNAi transgenic lines (Figure 2) and it was induced by multiple hormones (SA, MeJA, ACC, and ABA) (Figure S3), indicating that its regulation is complex and multifactorial.

Heterologous overexpression of *PgPR10‐1* in *Arabidopsis* conferred enhanced resistance to *Pseudomonas syringae* , *Fusarium oxysporum*, and *B. cinerea* (Lee et al. 2012). ScPR10 was able to enhance the resistance of *N. benthamiana* leaves to infections by *Pseudomonas solanacearum* and *Fusarium solani* var. *coeruleum* (Peng et al. 2017). However, overexpression of the pea *PR10.1* gene in canola did not significantly enhance resistance to pathogens, suggesting that the function of *PR10* genes may be influenced by plant species and the type of pathogens involved.

In this study, overexpression of *FvPR10.14* significantly reduced the number of *P. aphanis* spores and enhanced the accumulation of ROS (H_2_O_2_ and O_2_.^−^) (Figure 6), indicating that *FvPR10.14* inhibits *P. aphanis* through several defence‐related pathways. The classic Bet_V1 domain of FvPR10.14 is likely involved in directly degrading the pathogen's nucleic acids. In addition, the ROS burst may act as a systemic signal during plant immunity responses that activate multiple stress‐responsive pathways and contribute to limiting pathogen spread. Similar mechanisms have been reported in grape *VpPR10*.*1*, and tea *CsPR10‐9* (Ma et al. 2018; Tao et al. 2025). It is worth noting that *FvPR10.14* was highly expressed in strawberry fruit at the late developmental stage (Figure 4B), suggesting that it may have a unique function in fruit disease resistance. This is consistent with the conclusion of Orozco‐Navarrete et al. (2021) that Fra a 1.02 regulates fruit defence. Silencing of *Fra a 1.02* altered the expression of transport genes glutathione‐S‐transferases (GSTs) and *WRKY33* (Orozco‐Navarrete et al. 2021).

In this study, we found that SA and *P. aphanis* activated *FaMYB63* transcription, and silencing of *FaMYB63* significantly reduced SA accumulation caused by *P. aphanis* infection (Figure 1), suggesting an essential role for FaMYB63 in response to SA signalling and powdery mildew resistance.

Plant hormones play a critical signalling role in plant disease resistance pathways. When pathogens invade a host plant, changes in hormone levels are triggered and transmitted to the nucleus through signal transduction (Denancé et al. 2013). SA plays a central role in systemic acquired resistance (SAR), particularly against biotrophic pathogens (Spoel and Dong 2024). This process activates transcriptional regulatory networks related to plant disease resistance and defence, leading to a series of physiological responses. Consequently, it is widely recognised that genes involved in disease resistance and defence are also responsive to plant hormone induction (Noman et al. 2019). Although some regulatory components involved in SA‐induced powdery mildew resistance have been identified in *Arabidopsis* and other crops, such as melon, apple, and grape (Wang, Wang, et al. 2023; Wang, Wu, et al. 2023; Lan et al. 2025; Feng et al. 2020), the underlying molecular regulatory mechanisms remain unclear in strawberry. R2R3 MYB TFs have critical roles in plant responses to a wide range of abiotic and biotic stresses, including drought, salinity, and pathogen infection (Wang, Niu, and Zheng 2021; Yu et al. 2023). Previous studies also indicated that MYB TFs function as critical nodes in SA signalling and phenylpropanoid defence metabolism (Feng et al. 2020; Zhu et al. 2021).

*MdMYB73* overexpression confers resistance to *Botryosphaeria dothidea* in apple ( *Malus domestica* ) calli and fruit by boosting the accumulation of SA and the expression of SA synthesis and signalling genes (Gu et al. 2021). CsMYB96, a *Citrus sinensis* homologue of *Arabidopsis* MYB96, directly activates the expression of *CsCBP60g*, thereby enhancing SA biosynthesis and strengthening host resistance to *Penicillium italicum* and *B. cinerea* (Zhang, Wang, et al. 2021). *PbrMYB14*‐overexpressing pears are more resistant to *Alternaria alternata* via *PbrPAL1* activation and an increase in SA concentration (Yan et al. 2025). However, although GhMYB36 enhances resistance to Verticillium wilt and drought by up‐regulating *PR1*, its expression is not induced by SA (Liu et al. 2022).

Our previous study identified FaMYB63 as a key regulator of eugenol biosynthesis during strawberry fruit ripening (Wang, Shi, et al. 2022). In this study, the decrease in SA concentration in the FaMYB63‐RNAi transgenic lines, and the induction of *FaMYB63* expression by SA treatment (Figure 1B–D,F), implies that FaMYB63 could participate in the dynamic balance of SA signalling that promotes plant resistance to the pathogen causing powdery mildew diseases. Silencing of *FaMYB63* resulted in a marked reduction in SA levels, significant down‐regulation of *FvICS1* and *FvPAL2*, and increased susceptibility to the pathogen. These findings suggest that FaMYB63 may be involved in SA‐dependent defence responses.

Interestingly, despite the reduced SA concentration, the expression of SA‐related defence markers such as *FvPR1*, *FvPR2‐1*, and *FvPR5‐2* was up‐regulated in FaMYB63‐RNAi transgenic lines (Figure S2). This paradoxical observation may reflect a compensatory response triggered by pathogen attack or activation of alternative defence signalling pathways, such as those mediated by JA or ROS. However, this up‐regulation appears insufficient to restore effective resistance in the absence of adequate SA accumulation. Together, these results indicate that elevated PR gene expression does not necessarily confer enhanced resistance and highlight the essential role of MYB63‐mediated SA biosynthesis in orchestrating an effective immune response. However, the mechanism by which FaMYB63 regulates SA biosynthesis and fine‐tunes the expression of resistance‐related genes in response to *P. aphanis* infection requires further investigation.

The *FvPR10.14* gene was also significantly up‐regulated by SA (Figure 5 and S3). Our results suggest that the FaMYB63‐*PR10.14* module may be a key regulatory step downstream of the SA signalling pathway, inhibiting *P. aphanis* infection by coordinating SA‐dependent defence responses. In wheat, TaMYB391 positively regulates resistance to stripe rust (*Puccinia striiformis*) by inducing PR genes, enhancing ROS accumulation, and triggering hypersensitive cell death possibly through SA signalling pathways (Hawku et al. 2022). TFs regulate the expression of their target genes by binding to specific DNA sequences in their promoters, thereby modulating their expression levels (Zhu et al. 2022; Cheng et al. 2016). Our findings suggest that *FvPR10.14* acts as a downstream regulatory target of FaMYB63 and WRKY75, linking MYB/WRKY‐mediated transcriptional control to PR10 function (Figures 3 and 8). The involvement of MYB and WRKY TFs in pathogen‐induced gene expression has been extensively documented. R2R3‐MYB transcription factor TaRIM1 in wheat enhances sharp eyespot resistance by binding to the promoter of the *PR10* gene (Shan et al. 2016). Ectopic expression of peanut *AhGLK1b* in *Arabidopsis* activates expression of *PR10* and *Phox/Bem 1* (PBI), conferring resistance to fungal and bacterial pathogens (Ali et al. 2020). In woody tea, silencing of *CsMYB72* increased SA concentrations and enhanced *Colletotrichum gloeosporioides* resistance. CsMYB72 also binds to and represses the *CsPR10‐9* promoter (Tao et al. 2025). OsWRKY67 positively regulates rice immunity against leaf blast, panicle blast, and bacterial blight by directly activating *PR10* and *PR1a* gene expression (Liu et al. 2018). CsWRKY25 and CsWRKY65 contribute to disease resistance of citrus fruit to *Penicillium digitatum* by directly activating the expression of *CsRbohB*, *CsRbohD*, and *CsPR10* (Wang, Li, et al. 2022, 2021). SlWRKY30 and SlWRKY81 synergistically decrease tomato susceptibility to *Ralstonia solanacearum* infection by directly activating the transcription of *SlPR‐STH2* genes (Dang et al. 2023).

**FIGURE 9:**
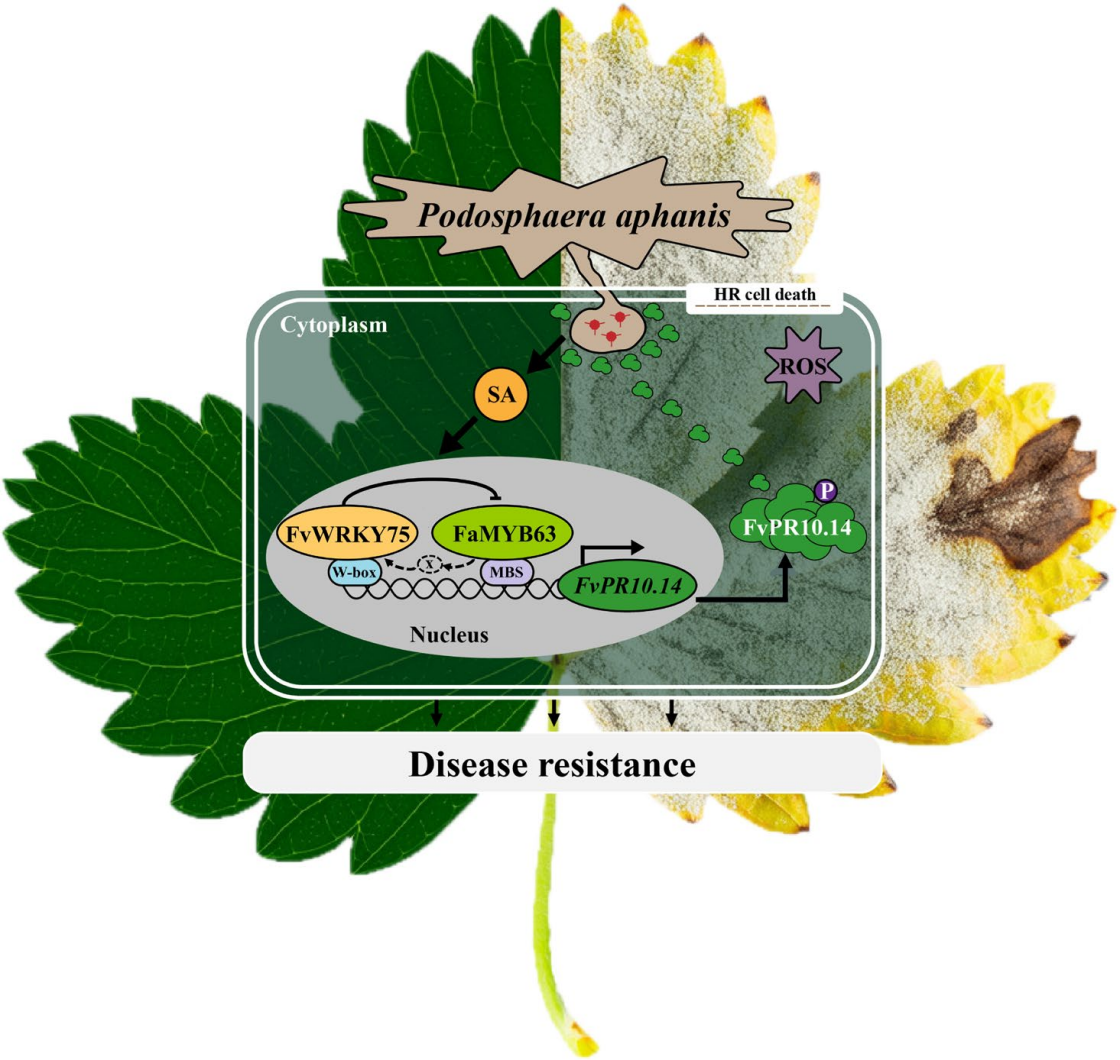
A proposed model illustrating the enhancement of strawberry resistance to *Podosphaera aphanis* mediated by MYB63 and WRKY75. Dashed arrows and ‘x’ indicate hypothetical or unverified regulatory relationships. HR, hypersensitive response; MBS, MYB binding site; ROS, reactive oxygen species; SA, salicylic acid.

Several studies have shown that WRKY TFs can take part in plant defence regulation controlled by MYB TFs. In *Arabidopsis*, AtMYB44 positively regulates SA‐mediated defence responses by activating *AtWRKY70*, thereby improving resistance to *P. syringae* pv. *tomato*. Conversely, AtMYB44 negatively regulates JA‐mediated defence gene expression, reducing resistance to *Alternaria* (Shim et al. 2013). In FaMYB63‐RNAi transgenic strawberry plants, *FvWRKY75* transcripts were significantly reduced. FvWRKY75 shares high amino acid sequence similarity with FaWRKY1 (Figure 7). Overexpression of *FaWRKY1* in WT *Arabidopsis* plants confers enhanced resistance to *P. syringae* (Encinas‐Villarejo et al. 2009). These findings suggest that FaMYB63 may enhance strawberry resistance to powdery mildew by regulating *FvWRKY75* expression. However, WRKY75 suppressed the expression of *MYB63* through a mechanism that does not require direct interaction between the two TFs (Figures 9 and S8). MYB21, MYB99, and MYB24 are involved in primary and phenylpropanoid metabolic pathways during anther development. *myb99* knockout mutation notably led to down‐regulation of *MYB21* expression. Promoter activation assays showed that both MYB99 and MYB21 are able to activate the promoters of *TK2*, a key gene involved in pollen coat formation. Interestingly, MYB21 repressed the promoter of *MYB99*, while activating that of *MYB24*. Together, these TFs form the MYB99‐MYB21‐MYB24 triad, which coordinately regulates *TK2* expression and the associated metabolic network in anthers (Battat et al. 2019). Therefore, we speculate that FaMYB63 may regulate the expression of *FvWRKY75* through other TFs to regulate strawberry resistance to powdery mildew, but the detailed molecular mechanism still requires further investigation.

In summary, our findings demonstrate that overexpression of *FvPR10.14* in strawberries enhances resistance to *P. aphanis*, possibly by altering the SA‐mediated defence response signalling network. FaMYB63 and FvWRKY75 are likely involved in the regulation of *FvPR10.14* transcription that enhances disease resistance (Figure 9). This study elucidates the molecular mechanisms underpinning strawberry's resistance to powdery mildew and identifies FaMYB63 and FvWRKY75 as promising targets for genetic breeding strategies aimed at strengthening disease resilience. Future research should clarify the roles of FaMYB63 and FvWRKY75 in defence mechanisms and the molecular roles of MYB and WRKY TFs in signalling crosstalk between SA and other hormones in immune signalling responses.

## 4 Experimental Procedures

### 4.1 Plant Material and Growth Condition

In this study, three FaMYB63‐RNAi transgenic lines were used, with untransformed WT *F. vesca* ‘Ruegen’ plants as the control. The strawberry, *N. benthamiana*, and *Arabidopsis thaliana* Col‐0 plants were grown in a greenhouse (20°C–25°C, 16 h/8 h light/dark photoperiod, 70%–85% relative humidity) at Anhui Agricultural University in 1 L pots with substrate containing vermiculite:perlite:worm castings (vol:vol:vol, 1:1:1). Fruit samples at seven different developmental stages: small green (SG, 7 days post‐anthesis [DPA]), large green (LG, 15 DPA), green‐white (GW, 20 DPA), white (Wt, 23 DPA), turning (T, 26 DPA), red‐ripening (R, 28 DPA), and over‐ripening (OR, 32 DPA) fruit stages and different organ tissues were collected from five or six individual plants. All fruits and tissues were washed with ultrapure water, then immediately frozen in liquid nitrogen and stored at −80°C.

### 4.2 Hormone Concentration Determination

SA concentrations in FaMYB63‐RNAi strawberry transgenic lines and WT were measured using ultra‐performance liquid chromatography‐mass spectrometry (UPLC‐MS). For each transgenic line and the WT, three biological replicates were prepared, each consisting of six leaves at similar developmental stages. A total of 100 mg of finely ground sample was weighed into a centrifuge tube containing zirconia beads. Extraction was performed with 1 mL of acetonitrile:water (1:1, vol/vol) containing a small amount of sodium diethyldithiocarbamate as an antioxidant. The mixture was extracted on ice for 4 h and then centrifuged at 12,900 *g* for 10 min at 4°C. The supernatant was collected for purification. Solid‐phase extraction (SPE) was carried out using a Waters MAX cartridge, which was preconditioned sequentially with 4 mL of methanol and 2 mL of 0.1 M ammonium hydroxide. The extract was evaporated to dryness under vacuum and reconstituted in 2 mL of 0.1 M ammonium hydroxide prior to loading onto the cartridge. The column was washed with 2 mL of 0.1 M ammonium hydroxide followed by 2 mL of 60% methanol in 0.1 M ammonium hydroxide. The analytes were eluted with 0.2 mL of methanol and the eluate was subjected to analysis.

Chromatographic separation was conducted using an ACQUITY UPLC system (Waters Corp.) equipped with an HSS T3 column (50 × 2.1 mm, 1.8 μm; Waters). The injection volume was 2 μL and the column temperature was maintained at 40°C. The mobile phase consisted of (A) 0.1% acetic acid in acetonitrile and (B) 0.1% acetic acid in water, operated under gradient conditions.

Mass spectrometric detection was performed on a Q Exactive high‐resolution mass spectrometer (Thermo Fisher Scientific) equipped with an electrospray ionisation (ESI) source operating in negative mode. Data were acquired in selected ion monitoring (SIM) mode to enhance sensitivity. The optimised MS parameters were as follows: sheath gas flow rate, 40 arbitrary units; auxiliary gas flow rate, 10 arbitrary units; spray voltage, −2800 V; capillary temperature, 350°C; and ion transfer tube temperature, 320°C. Quantification was carried out using external calibration with authentic standards. Standards of SA (69‐72‐7) were purchased from Sigma‐Aldrich.

### 4.3 RNA Extraction and RT‐qPCR Assays

RNA extraction and RT‐qPCR assays were performed using the methods as described by Wang, Shi, et al. (2022). Briefly, total RNA of collected samples was extracted using an RNA Extraction Kit (Genesand Biotech Co. Ltd). qPCR analysis was performed using Hieff qPCR SYBR Green Master Mix (High Rox Plus) (11203ES03, Yeasen) under the following cycling procedure: 40 cycles of 95°C for 10 s and 60°C for 30 s in a Step One Plus Real‐Time PCR system instrument (Applied Biosystems). RT‐qPCR analysis was performed using three independent biological replicates, each with three technical replicates. The relative RNA abundance was calculated using the 2^−ΔΔ*C*t^ method, with *interspacer 26S‐18S* as the housekeeping sequence. All primers used for the experiments are listed in Table S1.

### 4.4 Sequence Analysis of FvPR10.14

The conserved domain of FvPR10.14 was predicted with STMART (https://smart.embl.de/) and Pfam (http://pfam‐legacy.xfam.org/). *cis*‐acting elements in the promoters of *FvPR10.14* were predicted using PlantCARE (http://bioinformatics.psb.ugent.be/webtools/plantcare/html/) and PLACE (http://www.dna.affrc.go.jp/PLACE/) databases. The nuclear localisation signal (NLS) region was predicted with a cNLS Mapper (http://nls‐mapper.iab.keio.ac.jp/cgi‐bin/NLS_Mapper_form.cgi). DNAMAN was used to perform multiple sequence alignments. A phylogenetic tree of the amino acid sequences was generated using MEGA 11 software with 1000 bootstrap replicates. The amino acid sequences used for phylogenetic tree construction are listed in Table S2.

### 4.5 Plasmid Construction and Genetic Transformation

The coding sequence of *FvPR10.14* was amplified and inserted into the pCAMBIA1302‐GFP vector to overexpress *FvPR10.14* under the 35S promoter. Ruegen plants were transformed with these vectors using *A. tumefaciens* GV3101–mediated transformation as previously described by Wang, Shi, et al. (2022). The 1996 bp promoter of *FvPR10.14* was cloned into the GUS expression vector pCAMBIA1391 to construct the P*FvPR10.14*‐GUS fusion vector, which was introduced into the *A. tumefaciens* GV3101 via heat shock transformation. *Arabidopsis* plants were transformed using the floral dip method. RT‐qPCR was used to analyse the expression levels of genes in the transgenic plants. Primers are listed in Table S3.

### 4.6 GUS Staining and Dual‐LUC Assays

To assess GUS activity, the 1996 bp and 1369 bp promoter regions of *FvPR10.14* and *FvWRKY75* were cloned into the pCAMBIA1391Z vector. The coding sequences of *FaMYB63* and *FvWRKY75* were inserted into the pGreenII 62‐SK vector to generate effector constructs. The resulting plasmids were introduced into *N. benthamiana* leaves via *A. tumefaciens* ‐mediated transient expression. The empty pGreenII 62‐SK vector was used as a negative control. Histochemical GUS staining was performed using X‐Gluc (5‐bromo‐4‐chloro‐3‐indolyl‐β‐D‐glucuronic acid), and the GUS enzymatic activity was quantified using a 4‐methylumbelliferone (Sigma‐Aldrich) standard curve. Both staining and quantification were repeated three times.

For dual‐LUC assays, *FvPR10.14* and *FaMYB63* promoter fragments (1996 bp and 1913 bp) were cloned into the pGreenII 0800‐LUC vector to generate reporter constructs. The 35S::REN cassette in the same vector was used as an internal control to normalise transfection efficiency. Effector (pGreenII 62‐SK‐*FaMYB63* or *FvWRKY75*) and reporter plasmids were individually introduced into *A. tumefaciens* GV3101 and co‐infiltrated into *N. benthamiana* leaves. LCU activity was measured 3 days post‐infiltration, and promoter activity was expressed as the LUC/REN ratio. Each treatment was repeated with at least nine biological replicates. Primers are listed in Table S3.

### 4.7 Y1H Assay and EMSA

For the Y1H assay, a 1996 bp fragment upstream of the *FvPR10.14* coding region was amplified by PCR and inserted into the pAbAi vector to generate the bait construct. The promoter sequence of *FaMYB63* was divided into two fragments (P1:−1 to −972 bp; P2: −917 to −1913 bp). Each of the two fragments was separately recombined into the pAbAi vector. Full‐length cDNAs of *FaMYB63* and *FvWRKY75* were cloned into the pGADT7 vector to generate prey constructs. Linearised bait plasmids were transformed into Y1HGold yeast strains using the Yeastmaker Yeast Transformation System 2 (Clontech), following the manufacturer's protocol. After determining the minimal inhibitory concentration of aureobasidin A (AbA) for each bait strain, one‐by‐one interactions between bait and prey constructs were assessed on SD/−Leu medium supplemented with AbA. The empty pGADT7 vector was used as a negative control. Primers are listed in Table S3.

EMSAs were performed according to the previous study of Wang, Shi, et al. (2022). The coding sequence (CDS) regions of *FaMYB63* and *FvWRKY75* were cloned into the pGEX4T‐1 vector to generate GST and His‐tagged fusion proteins, respectively. These constructs were transformed into *Escherichia coli* BL21 (DE3), and expression was induced with 0.5 mM IPTG. The recombinant proteins were purified using GSTrap FF columns (GE Healthcare). EMSAs were conducted with the EMSA Kit (Beyotime). The primers are presented in Table S3.

### 4.8 Y2H and Split‐LUC Assays

Y2H assays were performed using the Matchmaker Gold Yeast Two‐Hybrid System (Clontech) according to the manufacturer's instructions. The EcoRI and SalI restriction enzymes were used to cleave the pGBKT7 vector, and the coding sequence of *FvWRKY75* was inserted into it and introduced into the yeast strain Y2HGold to generate FvWRKY75‐BD bait. The BamHI and EcoRI restriction enzymes were used to cleave the pGADT7 plasmid, and the full length of *FaMYB63* was recombined into it using homologous ligation to generate FaMYB63‐AD. The resulting plasmids FvWRKY75‐BD and FaMYB63‐AD were co‐transferred into the yeast strain Y2H‐Gold, and monoclonal clones selected from SD/−Trp−Leu medium were inoculated to SD/−Trp−Leu−His−Ade and SD/−Trp−Leu−His−Ade+X‐α‐Gal media (Coolaber). The primers are listed in Table S3.

The CDS regions of *FaMYB63* and *FvWRKY75* were cloned into JW771 and JW772 to obtain FvWRKY75‐nLUC and FaMYB63‐cLUC, respectively. The recombinant plasmids were co‐transformed into *A. tumefaciens* GV3101, and the activated agrobacteria were resuspended to an OD_600_ of 1.2. The combination was infiltrated into varying positions of the same leaf of *N. benthamiana*. After 3 days, the transfected *N. benthamiana* leaves were analysed using a 5200 multichemiluminescence imaging system (Tanon). The primers are listed in Table S3.

### 4.9 DAB, NBT, and Trypan Blue Stain

DAB, NBT, and trypan blue staining were performed as previously described by Li et al. (2023). Trypan blue staining was used to assess cell death. Leaf samples at various time points post‐inoculation were immersed in trypan blue solution and boiled for staining. Strawberry leaves were boiled for 5 min, followed by destaining with chloral hydrate solution.

DAB staining was used to detect H_2_O_2_ accumulation. Leaves were immersed in DAB solution and incubated for 8 h, then destained three times with 95% ethanol.

NBT staining was performed to visualise superoxide anion (O_2_.^−^) accumulation. Leaves were immersed in NBT solution for 8 h after inoculation, then soaked in 80% ethanol and destained at 60°C for 2 h. The DAB and NBT staining areas were quantified using ImageJ (Fiji) software (Sekulska‐Nalewajko et al. 2016). Three independent biological replicates were collected for each transgenic line and the WT.

### 4.10 Subcellular Localisation of FvPR10.14

The 35S::*FvPR10.14*‐GFP construct was transformed into *A. tumefaciens* (pSoup‐p19) and infiltrated into *N. benthamiana* leaves as described previously (Wang, Shi, et al. 2022). Fluorescence signals were checked with an epifluorescence microscope (Olympus). The fluorescence wavelength of GFP was detected at 480 to 520 nm.

### 4.11 Hormone Treatments

Hormone treatments were performed based on the method described by Wei et al. (2017), with minor modifications. Uniform in vitro‐grown seedlings of Ruegen bearing 10 leaves were selected for the experiment. Murashige and Skoog (MS) medium was prepared and autoclaved at 116°C for 30 min, then cooled to 55°C in an oven. In a sterile laminar flow hood, appropriate volumes of stock solutions of SA, MeJA, ACC, and ABA were added to the medium to achieve a final concentration of 0.1 mM for each hormone. The media were mixed thoroughly and allowed to cool to room temperature.

Sterile forceps were used to transfer the aseptic seedlings onto hormone‐supplemented media. Leaf samples were collected at 0, 0.5, 2, 4, 8, 12, 24, 48, and 72 h post‐treatment. Each treatment was performed with three biological replicates.

### 4.12 *Podosphaera aphanis* Inoculation and Quantification of Pathogen Biomass

*Podosphaera aphanis* isolates were collected from naturally infected strawberry leaves in the strawberry germplasm resource greenhouse at Anhui Agricultural University. The isolates were propagated on *F. vesca* (cv. Ruegen) following the method described by Hu et al. (2015) and Tapia et al. (2021), to establish a stable inoculum source for subsequent experiments.

For formal inoculation, 6‐month‐old strawberry plants with 10 fully expanded leaves were used. The experimental layout followed a randomised complete block design with three independent biological replicates per line (WT and FaMYB63‐RNAi#1, #2, and #3). Plants were maintained under controlled conditions: 22°C, approximately 85% relative humidity, and a photoperiod of 8 h light/16 h dark. The first fully expanded leaves were collected at 0, 4, 8, 12, 24, 48, 72, and 120 h post‐inoculation (hpi) and 7 days post‐inoculation (dpi). Approximately 200 mg of infected leaf tissue was immersed in 200 mL of sterile 10% glycerol solution in a 5 mL centrifuge tube and vortexed for 5 min. The spore suspension was then loaded onto a haemocytometer, and the conidia were counted under a microscope (BX63, Olympus). The data were obtained from three infected leaves. Samples were immediately frozen in liquid nitrogen and kept at −80°C until further analysis. Each sample was analysed in triplicate.

### 4.13 Statistical Analysis

The data was statistically analysed by the SPSS v. 19.0 software. All data are presented as the mean ± SD of three or nine biological replicates. Statistical analysis was performed by one‐way ANOVA with Tukey HSD using SPSS v. 19.0. Student's *t* test was used to compare between two groups (*p < 0.05, **p < 0.01, ***p < 0.001) (GraphPad Prism v. 8.0.2).

## Author Contributions

**Rongyi Jiang:** data curation, methodology, conceptualization, validation. **Tao Tao:** methodology, data curation, investigation. **Xingbin Xie:** writing – original draft, data curation, methodology. **Yang Liu:** methodology, investigation. **Yanan Sun:** investigation. **Yang Zhang:** investigation. **Guanghui Zheng:** investigation. **Peipei Sun:** investigation. **Mauren Jaudal:** writing – review and editing, investigation. **Simona Nardozza:** writing – review and editing, investigation. **Congbing Fang:** conceptualization, project administration, writing – review and editing, supervision. **Jing Zhao:** conceptualization, data curation, investigation, supervision, writing – original draft, writing – review and editing, project administration.

## Funding

This work was supported by the National Key R&D Program of China (grant no. 2022YFD1600700), the Natural Science Foundation of Anhui Province (grant no. 2508085MC057), Key Project of Natural Science Research for Colleges and Universities in Anhui Province (grant no. 2023AH051043) and the National Natural Science Foundation of China (grant no. 32472661).

## Conflicts of Interest

The authors declare no conflicts of interest.

## Supporting information


**FIGURE S1:** FaMYB63‐RNAi strawberry plants exhibit decreased resistance to powdery mildew. Disease phenotype of wild‐type (WT) and FaMYB63‐RNAi plants (RNAi#1, #2, and #3) at 7 days post‐inoculation (dpi) with *Podosphaera aphanis*. Scale bar = 5 mm.

**FIGURE S2:** Relative expression of salicylic acid (SA) biosynthesis‐related and pathogenesis‐related (PR) genes in FaMYB63‐RNAi plants (RNAi#1, #2, and #3). Data indicates mean ± standard deviation (SD) of three replicates relative to the housekeeping gene *interspacer 26S‐18S*. Student's *t* test: ***p < 0.001; **p < 0.01; *p < 0.05.

**FIGURE S3:** The expression profile of the *FvPR10s* in response to salicylic acid (SA), methyl jasmonate (MeJA), ethylene (ACC), and abscisic acid (ABA). (A–D) Expression levels of *FvPR10s* at different time points following SA (A), MeJA (B), ethylene (ACC) (C), and ABA (D) treatments. The colour scale represents relative expression levels, with red indicating increased transcript abundance and blue indicating decreased transcript abundance. Data indicates mean ± SD (*n* = 3).

**FIGURE S4:** *cis*‐element analysis of the *FvPR10.14* promoter.

**FIGURE S5:** Phylogenetic tree of FvPR10.14. Different colours represent different species.

**FIGURE S6:** Observation of GFP fluorescence in the FvPR10.14 ‐overexpressing and WT strawberry roots after 10 days of selection on selective media.

**FIGURE S7:** The expression of *FvWRKY75* is not directly activated by FaMYB63. Each treatment was performed in triplicate and each replicate contained 3 leaves. The representative photographs are shown here.

**FIGURE S8:** FvWRKY75 directly binds to *FaMYB63* promoter and suppresses its transcription. (A) Y1H assay between the interaction of FvWRKY75 and the promoters of *FaMYB63*. (B) Images of the luciferase and relative LUC/REN activity between the interaction of FvWRKY75 and the promoter of *FaMYB63*. Statistical significance was analysed using a Student's *t* test (***p < 0.001).

**FIGURE S9:** FaMYB63 can't interact directly with FvWRKY75. (A) A Split‐LUC assay indicated no interaction of FaMYB63 and FvWRKY75. (B) A Y2H assay indicated no interaction of FaMYB63 and FvWRKY75. X‐*α*‐gal, 5‐bromo‐4‐chloro‐3‐indolyl‐*α*‐D‐galactopyranoside; 3‐AT, 3‐amino‐1,2,4‐triazole.

**TABLE S1:** Primer sequences used for RT‐qPCR. fw: forward; rv: reverse.

**TABLE S2:** Subgroup classification of PR10 sequences examined in this study and additional information.

**TABLE S3:** Primer sequences used for PCR. fw: forward; rv: reverse.

## Data Availability

The data that supports the findings of this study are available in the Supporting Information of this article.
