# Supplementary material for: FaMYB63 and FvWYRKY75 Activate FvPR10.14 Boosting Strawberry Immunity Against Powdery Mildew
Source: Mol Plant Pathol. 2025 Dec 8;26(12):e70186. doi: 10.1111/mpp.70186 (PMC12686569; doi:10.1111/mpp.70186)
Supplement: Supplementary file 1 — FIGURE S1: FaMYB63‐RNAi strawberry plants exhibit decreased resistance to powdery mildew. Disease phenotype of wild‐type (WT) and FaMYB63‐RNAi plants (RNAi#1, #2, and #3) at 7 days post‐inoculation (dpi) with Podosphaera aphanis. Scale bar = 5 mm. [file MPP-26-e70186-s009.docx]

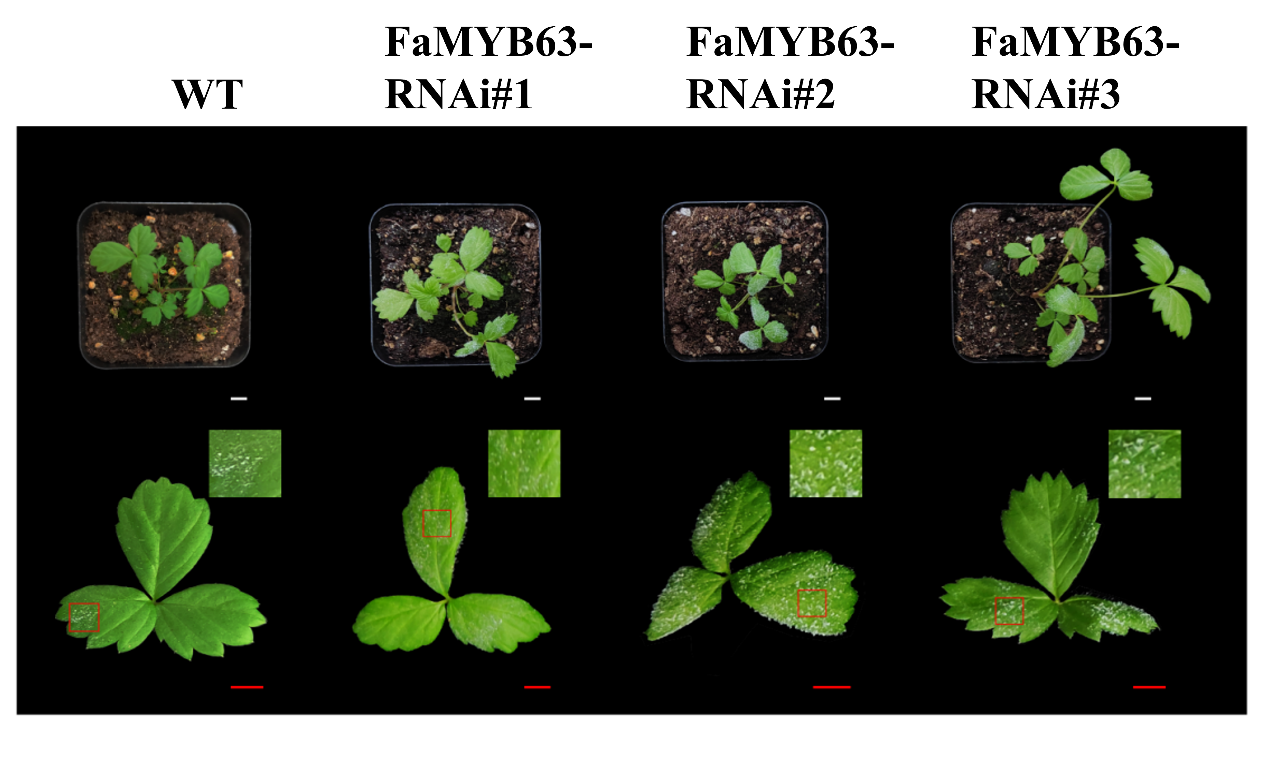


**FIGURE S1 | FaMYB63-RNAi strawberry plants exhibit decreased resistance to powdery mildew.**

Disease phenotype of wild-type (WT) and FaMYB63-RNAi plants (RNAi#1, #2, and #3) at 7 days post-inoculation (dpi) with *Podosphaera aphanis*. Scale bar = 5 mm.
