# Supplementary material for: FaMYB63 and FvWYRKY75 Activate FvPR10.14 Boosting Strawberry Immunity Against Powdery Mildew
Source: Mol Plant Pathol. 2025 Dec 8;26(12):e70186. doi: 10.1111/mpp.70186 (PMC12686569; doi:10.1111/mpp.70186)
Supplement: Supplementary file 2 — FIGURE S2: Relative expression of salicylic acid (SA) biosynthesis‐related and pathogenesis‐related (PR) genes in FaMYB63‐RNAi plants (RNAi#1, #2, and #3). Data indicates mean ± standard deviation (SD) of three replicates relative to the housekeeping gene interspacer 26S‐18S. Student's t test: ***p < 0.001; **p < 0.01; *p < 0.05. [file MPP-26-e70186-s004.docx]

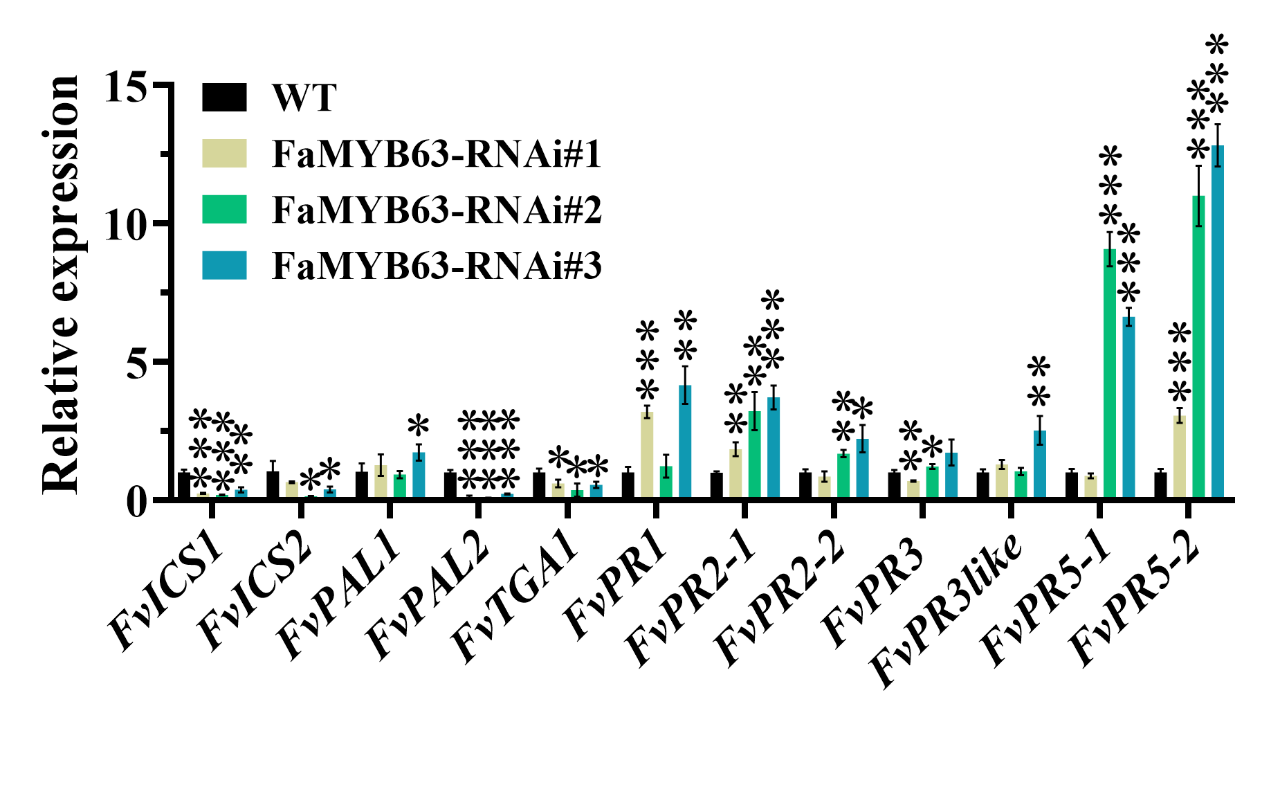


**FIGURE S2 | Relative expression of salicylic acid (SA) biosynthesis-related and pathogenesis-related (PR) genes in FaMYB63-RNAi plants (RNAi#1, #2, and #3).**

Data indicates mean ± standard deviation (SD) of three replicates relative to the housekeeping gene *interspacer 26S-18S*. Student’s *t* test: *** *P* < 0.001; ** *P* < 0.01: * *P* < 0.05.
