# Supplementary material for: FaMYB63 and FvWYRKY75 Activate FvPR10.14 Boosting Strawberry Immunity Against Powdery Mildew
Source: Mol Plant Pathol. 2025 Dec 8;26(12):e70186. doi: 10.1111/mpp.70186 (PMC12686569; doi:10.1111/mpp.70186)
Supplement: Supplementary file 3 — FIGURE S3: The expression profile of the FvPR10s in response to salicylic acid (SA), methyl jasmonate (MeJA), ethylene (ACC), and abscisic acid (ABA). (A–D) Expression levels of FvPR10s at different time points following SA (A), MeJA (B), ethylene (ACC) (C), and ABA (D) treatments. The colour scale represents relative expression levels, with red indicating increased transcript abundance and blue indicating decreased transcript abundance. Data indicates mean ± SD (n = 3). [file MPP-26-e70186-s001.docx]

**
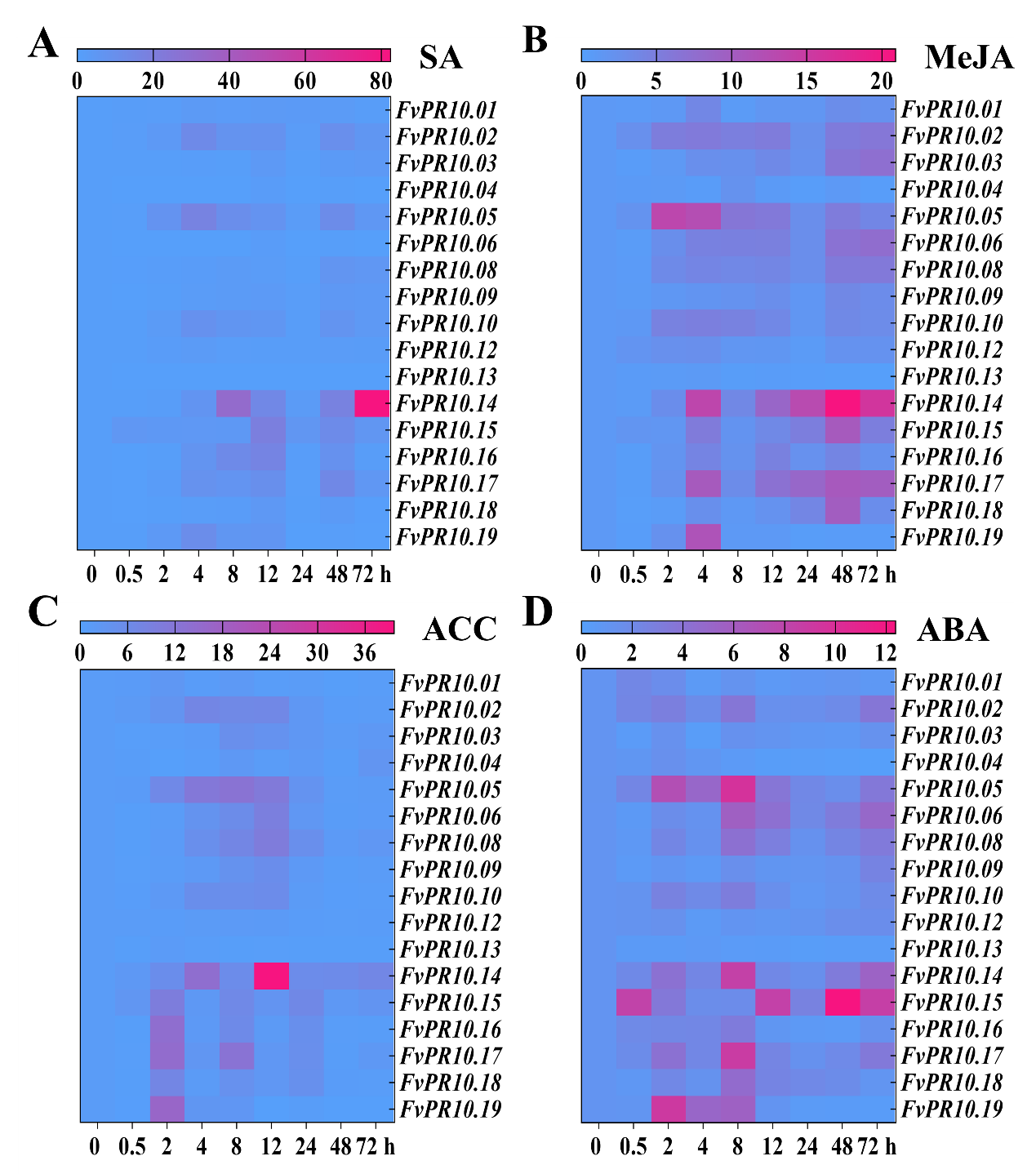
**

**FIGURE S3 | The expression profile of the *FvPR10s* in response to salicylic acid (SA), methyl jasmonate (MeJA), ethylene (ACC), and abscisic acid (ABA).**

(A-D) Expression levels of *FvPR10s* at different time points following SA (A), MeJA (B), ethylene (ACC) (C), and ABA (D) treatments. The color scale represents relative expression levels, with red indicating increased transcript abundance and blue indicating decreased transcript abundance. Data indicates mean ± SD (n = 3).
