# Supplementary figures and images for: FaMYB63 and FvWYRKY75 Activate FvPR10.14 Boosting Strawberry Immunity Against Powdery Mildew
Source: Mol Plant Pathol. 2025 Dec 8;26(12):e70186. doi: 10.1111/mpp.70186 (PMC12686569; doi:10.1111/mpp.70186)

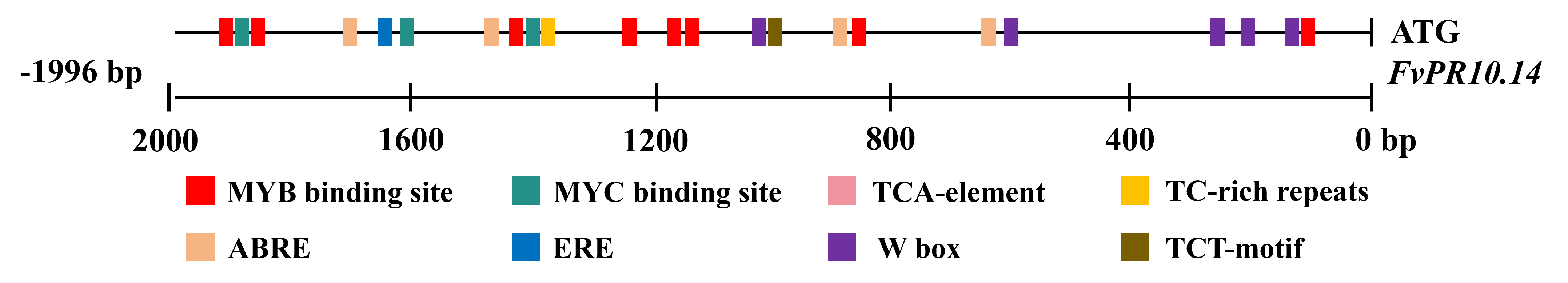


**FIGURE S4 | *Cis*-element analysis of the *FvPR10.14* promoter.**

Supplement: Supplementary file 4 — FIGURE S4: cis‐element analysis of the FvPR10.14 promoter. [file MPP-26-e70186-s007.docx]

**
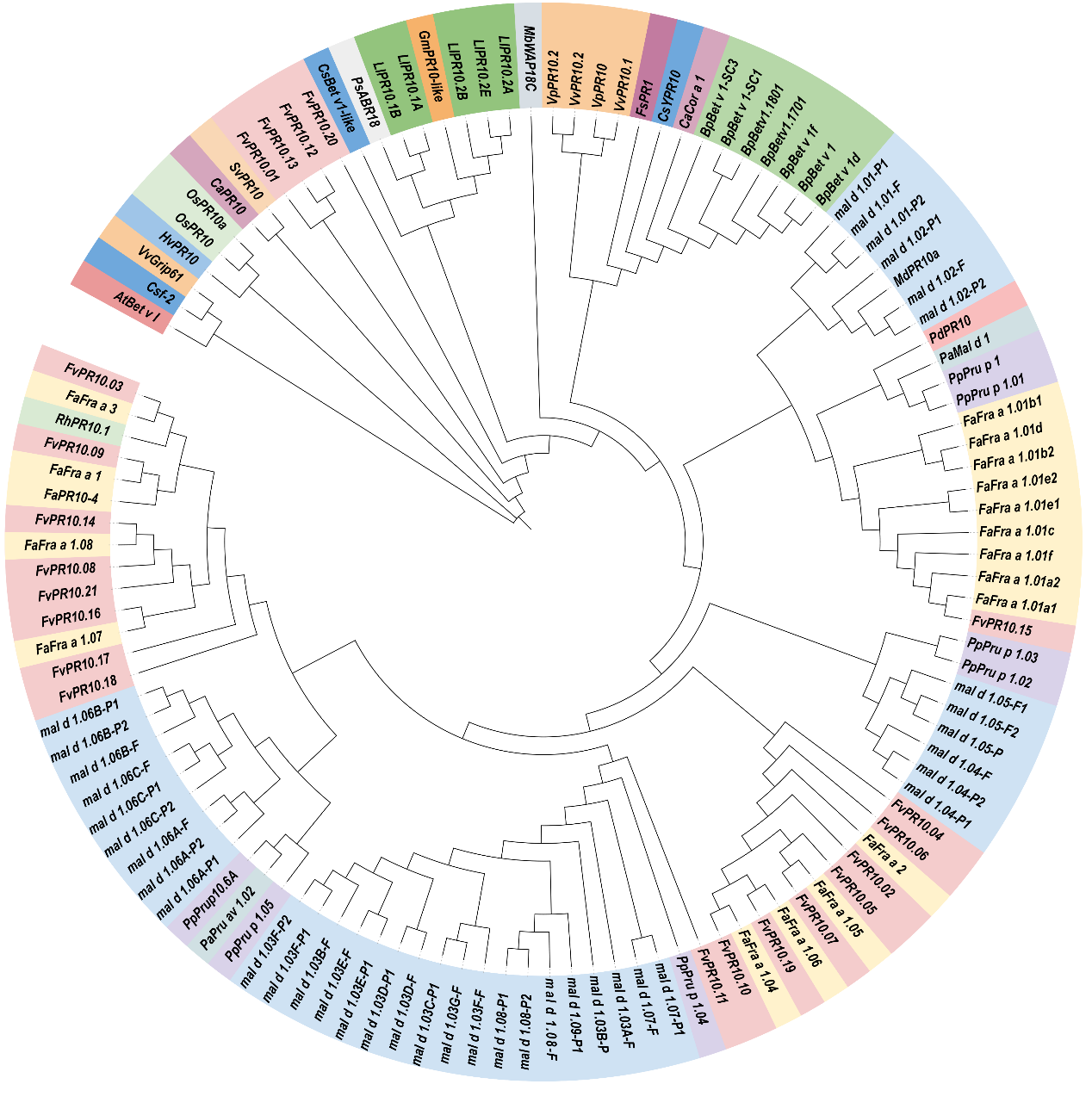
**

**FIGURE S4 | Phylogenetic tree of FvPR10.14.**

Different colours represent different species.

Supplement: Supplementary file 5 — FIGURE S5: Phylogenetic tree of FvPR10.14. Different colours represent different species. [file MPP-26-e70186-s010.docx]
