# Supplementary material for: FaMYB63 and FvWYRKY75 Activate FvPR10.14 Boosting Strawberry Immunity Against Powdery Mildew
Source: Mol Plant Pathol. 2025 Dec 8;26(12):e70186. doi: 10.1111/mpp.70186 (PMC12686569; doi:10.1111/mpp.70186)
Supplement: Supplementary file 6 — FIGURE S6: Observation of GFP fluorescence in the FvPR10.14 ‐overexpressing and WT strawberry roots after 10 days of selection on selective media. [file MPP-26-e70186-s003.docx]

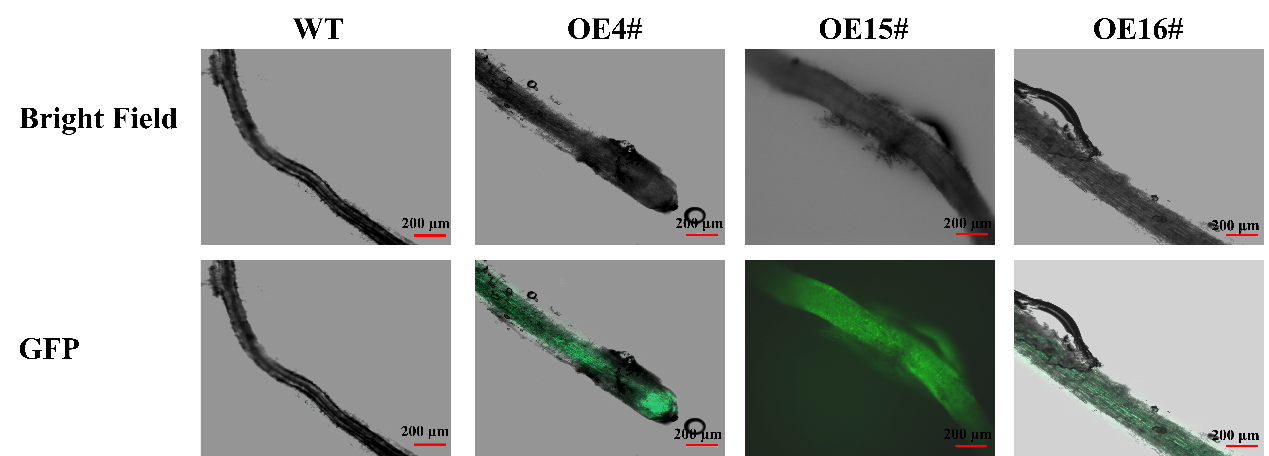


**FIGURE S6 Observation of GFP fluorescence in the FvPR10.14-overexpressing and WT strawberry roots after 10 days of selection on selective media.**
