# Supplementary material for: FaMYB63 and FvWYRKY75 Activate FvPR10.14 Boosting Strawberry Immunity Against Powdery Mildew
Source: Mol Plant Pathol. 2025 Dec 8;26(12):e70186. doi: 10.1111/mpp.70186 (PMC12686569; doi:10.1111/mpp.70186)
Supplement: Supplementary file 7 — FIGURE S7: The expression of FvWRKY75 is not directly activated by FaMYB63. Each treatment was performed in triplicate and each replicate contained 3 leaves. The representative photographs are shown here. [file MPP-26-e70186-s002.docx]

**
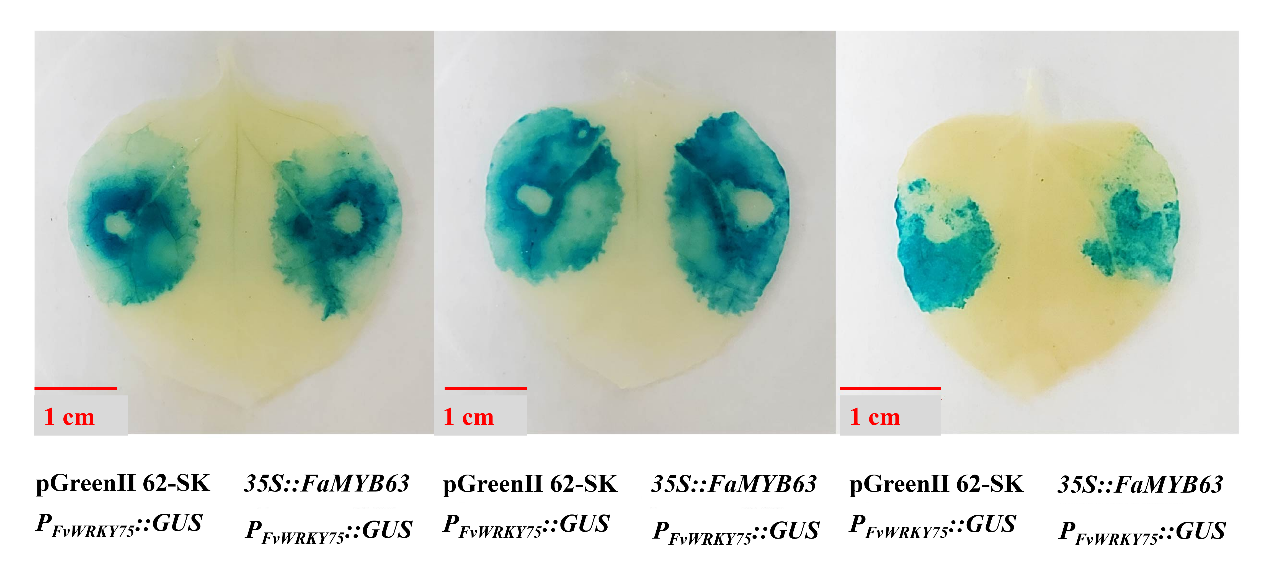
**

**FIGURE S7 | The expression of *FvWRKY75* is not directly activated by FaMYB63.**

Each treatment was performed in triplicate and each replicate contained 3 leaves. The representative photographs are shown here.
