# Supplementary material for: FaMYB63 and FvWYRKY75 Activate FvPR10.14 Boosting Strawberry Immunity Against Powdery Mildew
Source: Mol Plant Pathol. 2025 Dec 8;26(12):e70186. doi: 10.1111/mpp.70186 (PMC12686569; doi:10.1111/mpp.70186)
Supplement: Supplementary file 8 — FIGURE S8: FvWRKY75 directly binds to FaMYB63 promoter and suppresses its transcription. (A) Y1H assay between the interaction of FvWRKY75 and the promoters of FaMYB63. (B) Images of the luciferase and relative LUC/REN activity between the interaction of FvWRKY75 and the promoter of FaMYB63. Statistical significance was analysed using a Student's t test (***p < 0.001). [file MPP-26-e70186-s006.docx]

**
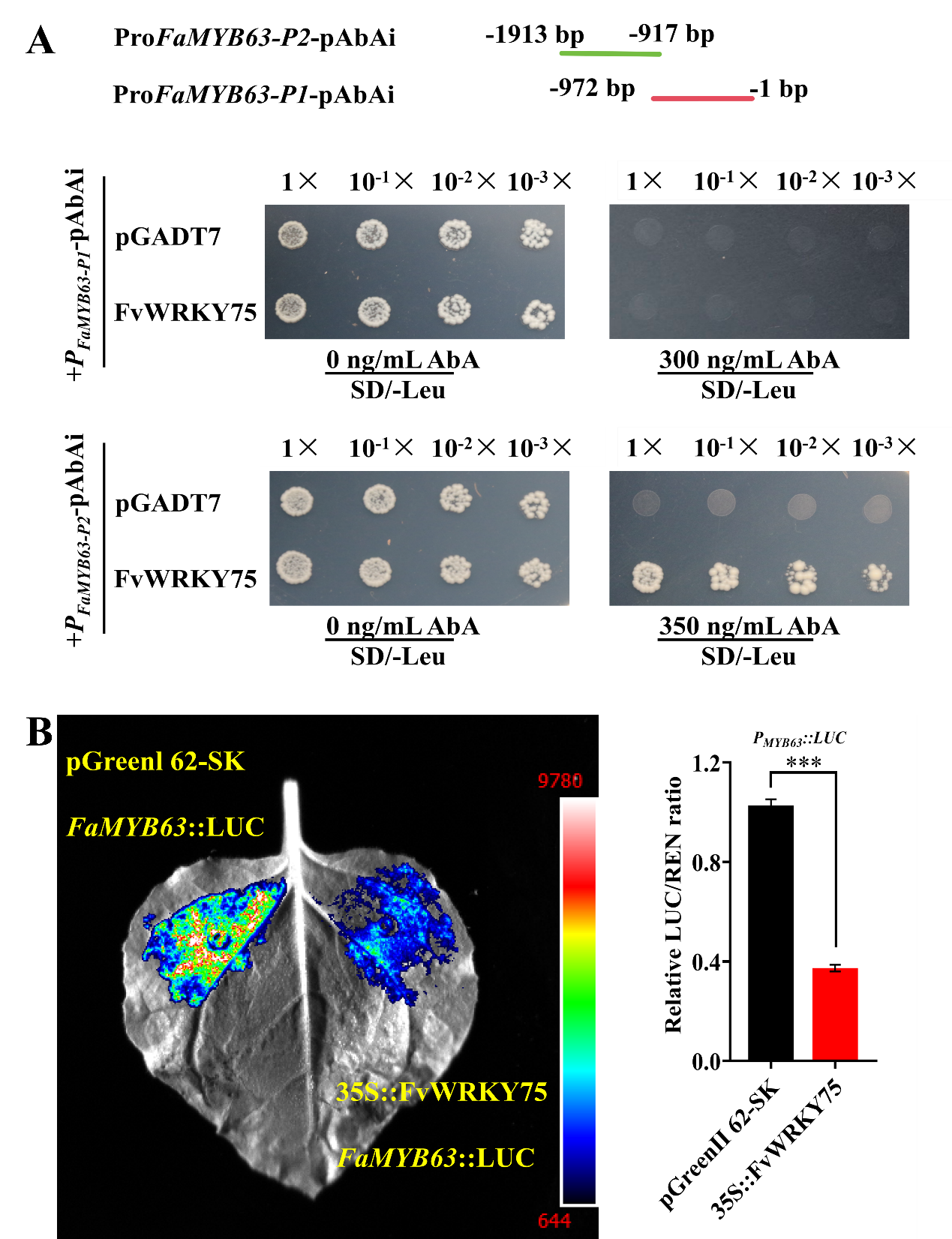
**

**FIGURE S8 | FvWRKY75 directly binds to *FaMYB63* promoter and suppresses its transcription.**

(A) Y1H assay between the interaction of FvWRKY75 and the promoters of *FaMYB63.* (B) Images of the luciferase and relative LUC/REN activity between the interaction of FvWRKY75 and the promoter of *FaMYB63*. Statistical significance was analyzed using a Student’s *t* test (****P* < 0.001).
