# Supplementary material for: FaMYB63 and FvWYRKY75 Activate FvPR10.14 Boosting Strawberry Immunity Against Powdery Mildew
Source: Mol Plant Pathol. 2025 Dec 8;26(12):e70186. doi: 10.1111/mpp.70186 (PMC12686569; doi:10.1111/mpp.70186)
Supplement: Supplementary file 9 — FIGURE S9: FaMYB63 can't interact directly with FvWRKY75. (A) A Split‐LUC assay indicated no interaction of FaMYB63 and FvWRKY75. (B) A Y2H assay indicated no interaction of FaMYB63 and FvWRKY75. X‐α‐gal, 5‐bromo‐4‐chloro‐3‐indolyl‐α‐D‐galactopyranoside; 3‐AT, 3‐amino‐1,2,4‐triazole. [file MPP-26-e70186-s012.docx]

**
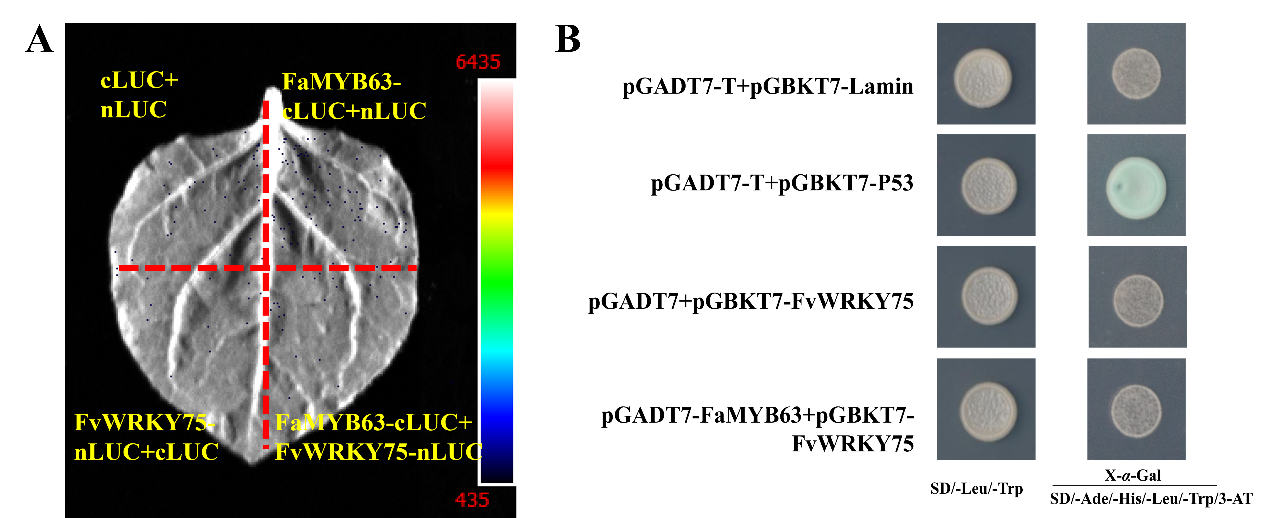
**

**FIGURE S9 |** **FaMYB63 can’t interact directly with FvWRKY75.**

1. A Split-LUC assay indicated no interaction of FaMYB63 and FvWRKY75. (B) A Y2H assay indicated no interaction of FaMYB63 and FvWRKY75. X-*α*-gal, 5-bromo-4-chloro-3-indolyl-α-D-galactopyranoside; 3-AT, 3-amino-1,2,4-triazole.
